# Optimization of hepatological clinical guidelines interpretation by large language models: a retrieval augmented generation-based framework

**DOI:** 10.1038/s41746-024-01091-y

**Published:** 2024-04-23

**Authors:** Simone Kresevic, Mauro Giuffrè, Milos Ajcevic, Agostino Accardo, Lory S. Crocè, Dennis L. Shung

**Affiliations:** 1 Department of Engineering and Architecture, University of Trieste, Trieste, Italy; 2 Department of Medicine (Digestive Diseases), Yale School of Medicine, Yale University, New Haven, CT USA; 3 Department of Medical, Surgical, and Health Sciences, University of Trieste, Trieste, Italy

**Keywords:** Translational research, Health policy

## Abstract

Large language models (LLMs) can potentially transform healthcare, particularly in providing the right information to the right provider at the right time in the hospital workflow. This study investigates the integration of LLMs into healthcare, specifically focusing on improving clinical decision support systems (CDSSs) through accurate interpretation of medical guidelines for chronic Hepatitis C Virus infection management. Utilizing OpenAI’s GPT-4 Turbo model, we developed a customized LLM framework that incorporates retrieval augmented generation (RAG) and prompt engineering. Our framework involved guideline conversion into the best-structured format that can be efficiently processed by LLMs to provide the most accurate output. An ablation study was conducted to evaluate the impact of different formatting and learning strategies on the LLM’s answer generation accuracy. The baseline GPT-4 Turbo model’s performance was compared against five experimental setups with increasing levels of complexity: inclusion of in-context guidelines, guideline reformatting, and implementation of few-shot learning. Our primary outcome was the qualitative assessment of accuracy based on expert review, while secondary outcomes included the quantitative measurement of similarity of LLM-generated responses to expert-provided answers using text-similarity scores. The results showed a significant improvement in accuracy from 43 to 99% (*p* < 0.001), when guidelines were provided as context in a coherent corpus of text and non-text sources were converted into text. In addition, few-shot learning did not seem to improve overall accuracy. The study highlights that structured guideline reformatting and advanced prompt engineering (data quality vs. data quantity) can enhance the efficacy of LLM integrations to CDSSs for guideline delivery.

## Introduction

Large language models (LLMs) have the potential to improve healthcare due to their capability to parse complex concepts and generate appropriate responses. LLMs have demonstrated proficiency in tasks across the spectrum of clinical activity, such as medical inquiry responses, dialogue systems, and the synthesis and completion of clinical reports^1–5^. One potential high-value area for LLMs is the ability to promote evidence-based practice through providing clinical decision support systems (CDSSs) according to current medical guidelines, which are distillations of both expert opinion and current evidence from clinical trials and are used to drive improvements in patient outcomes through best practices^6,7^.

LLMs have enjoyed wide public uptake, especially OpenAI’s ChatGPT (https://openai.com/blog/chatgpt), which enrolled over 100 million users within two months of its release^8,9^. The widespread use of ChatGPT allowed simple and user-friendly use of generative artificial intelligence for real-life scenarios and academic research. However, a primary concern for LLMs application in healthcare is the potential risk of inaccurate responses (e.g., “hallucinations”) that may lead to patient harm^10^. In clinical applications, a proposed framework for utilizing LLMs is based on adherence to the three principles of Honesty, Helpfulness, and Harmlessness (the HHH principle)^11^. To align LLMs to the HHH principle, specific strategies must be undertaken to bind their responses to a specific set of domain knowledge, such as retrieval augmented generation (RAG)^12^ or supervised fine-tuning (SFT) followed by Reinforcement Learning with Human Feedback (RLHF)^13^. Both RAG and SFT guide output generation according to a domain-specific dataset of information that, for clinical applications, could be represented by medical guidelines. However, the format of clinical guidelines is subject to broad variations (e.g., general structure, location of recommendations, table format, and flowcharts) that can affect the proper interpretation or retrieval of relevant information.

While the integration of LLMs in healthcare shows promise, the challenge of ensuring accurate interpretation of clinical guidelines becomes particularly relevant in the context of managing widespread chronic diseases such as Hepatitis C Virus (HCV) infection. New antiviral therapies successfully eradicate the disease, with multiple regimens demonstrating >90% efficacy and effectiveness^14^. HCV management has been codified in multiple guidelines that distill the results from the available randomized controlled trials to recommend best practices in chronic HCV diagnosis and treatment. However, adherence to guidelines ranges from 36–54% for screening and managing chronic HCV infection^15,16^. There is a need for scalable and reliable solutions to provide guideline-recommended care and bridge the gap in adherence, especially considering the World Health Organization’s goal to eliminate Hepatitis C by 2030^17^.

We present a novel LLM framework integrating clinical guidelines with RAG, prompt engineering, and text reformatting strategies for augmented text interpretation that significantly outperforms the baseline LLM model in producing accurate guideline-specific recommendations, with the primary outcome of qualitatively measuring accuracy based on manual expert review. We also apply quantitative text-similarity methods^18–21^ to compare the similarity of the LLM output to expert-generated responses.

## Results

### Output accuracy analysis

**Fig. 1:**
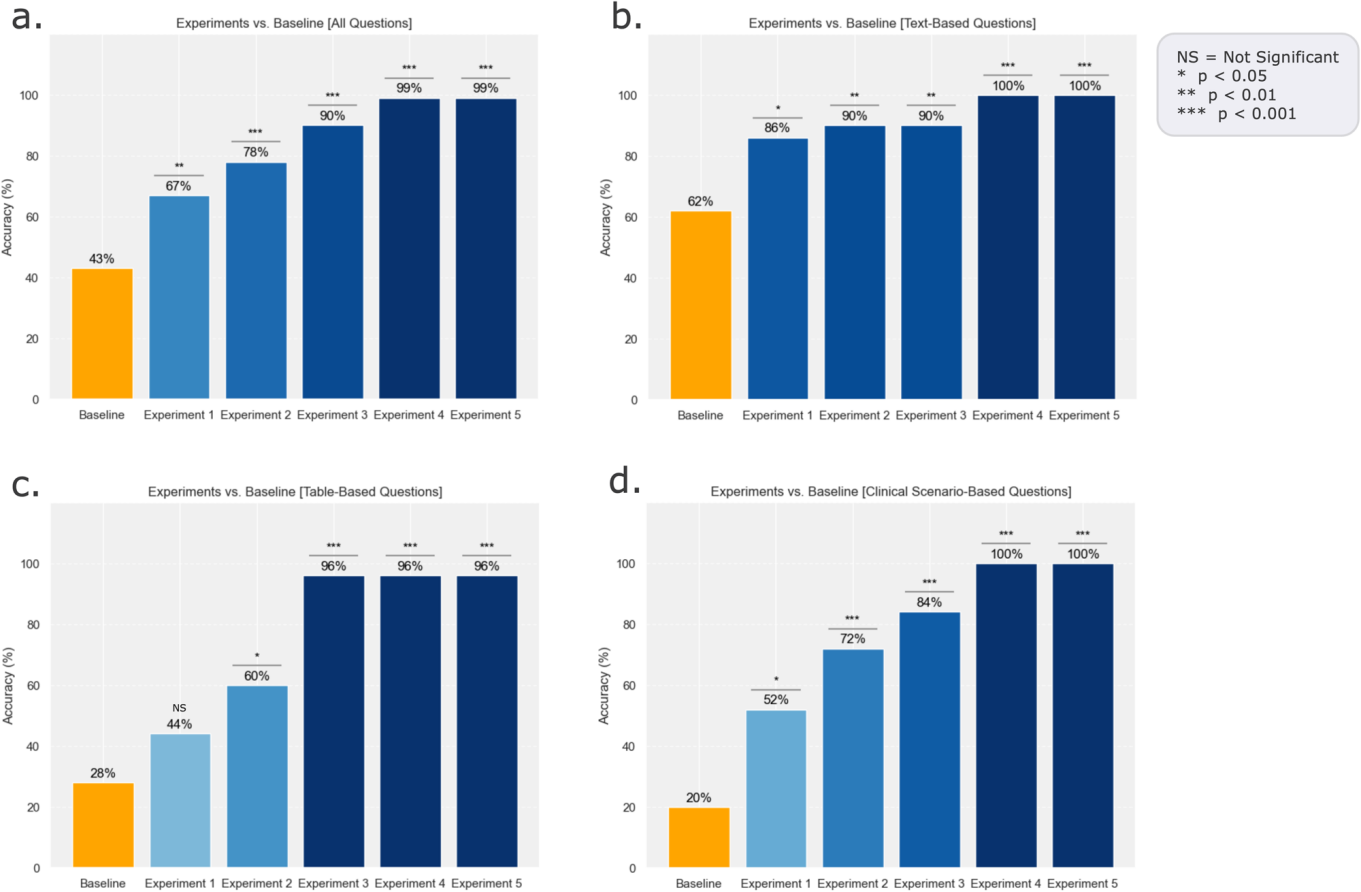
Qualitative evaluation of accuracy among all experiments from baseline. **a** Accuracy for all questions. **b** Accuracy only for text-based questions. **c** Accuracy for table-based questions. **d** Accuracy for clinical scenario-based questions. Statistical testing is based on pairwise comparison (Chi-Squared Test) between each experimental setting and the baseline.

The customized LLM framework achieved 99.0% overall accuracy, which was significantly better than the GPT-4 Turbo alone (99.0% vs. 43.0%; *p* < 0.001). Incorporating in-context guidelines improved accuracy (67.0% vs. 43.0%; *p* = 0.001). When the in-context guidelines were cleaned, and tables were converted from images to *.csv* files, accuracy improved to 78.0% (vs. 43.0%; *p* < 0.001); after the guidelines were formatted with a consistent structure and tables were re-formatted to text-based lists, accuracy further improved to 90.0% (vs. 43.0%; *p* < 0.001). Finally, the addition of custom prompt engineering led to an improvement in accuracy of 99.0% (vs. 43.0%; *p* < 0.001), with no further improvement despite few-shot learning with 54 question-answer pairs (Table 1, Fig. 1).

**Table 1:** Qualitative evaluation of accuracy based on human expert grading of each answer across all experimental settings

Metrics	Baseline	Experiment 1	Experiment 2	Experiment 3	Experiment 4	Experiment 5
All questions:						
Accuracy	43.0%	67.0%	78.0%	90.0%	99.0%	99.0%
Statistical Significance		*p* = 0.001	*p* < 0.001	*p* < 0.001	*p* < 0.001	*p* < 0.001
Text-based questions:						
Accuracy	62.0%	86.0%	90.0%	90.0%	100.0%	100.0%
Statistical significance		*p* = 0.012	*p* = 0.002	*p* = 0.002	*p* < 0.001	*p* < 0.001
Table-based questions:						
Accuracy	28.0%	44.0%	60.0%	96.0%	96.0%	96.0%
Statistical significance		*p* = 0.377	*p* = 0.046	*p* < 0.001	*p* < 0.001	*p* < 0.001
Clinical scenarios questions:						
Accuracy	20.0%	52.0%	72.0%	84.0%	100.0%	100.0%
Statistical significance		*p* = 0.039	*p* < 0.001	*p* < 0.001	*p* < 0.001	*p* < 0.001

Statistical testing is based on pairwise comparison (Chi-Squared Test) between each experimental setting and the baseline.

For text-based questions, the customized framework achieved 100% overall accuracy, which was better than GPT-4 Turbo alone (100% vs. 62.0%; *p* < 0.001). Incorporating in-context guidelines improved accuracy (86.0% vs. 62.0%; *p* = 0.01); after cleaning the text and conversion of tables from images to *.csv*, further improvement in accuracy was achieved with no further improvement after formatting the text into a consistent structure and converting tables into text-based lists (90.0% vs. 62.0%; *p* = 0.002). Adding custom prompt engineering resulted in 100% accuracy (100% vs. 62.0%; *p* < 0.001) with equivalent performance after few-shot learning with 54 question-answer pairs (100% vs. 62.0%; *p* < 0.001).

For table-based questions, the customized framework achieved 96.0% overall accuracy, which was better than GPT-4 Turbo alone (96.0% vs. 28.0%; *p* < 0.001). Incorporating in-context guidelines improved accuracy (44.0% vs. 28.0%; *p* = 0.38); after cleaning the text and conversion of tables from images to *.csv*, accuracy reached 60.0% (vs. 28.0%; *p* = 0.046) with a substantial improvement after converting tables into text-based lists and formatting the text into a consistent structure (96.0% vs. 28.0%; *p* < 0.001) with similar performance in Experiments 4 and 5 as reported in Table 1.

The customized framework achieved 100% overall accuracy for clinical scenarios, which was better than GPT-4 Turbo alone (100% vs. 20.0%; *p* < 0.001). Incorporating in-context guidelines improved accuracy (52.0% vs. 20.0%; *p* = 0.039); after cleaning the text and conversion of tables from images to *.csv*, accuracy reached 72.0% (vs. 20.0%; *p* < 0.001) with a substantial improvement after converting tables into lists and formatting the text into a consistent structure (84.0% vs. 20.0%; *p* < 0.001). Finally, the addition of custom prompt engineering achieved an accuracy of 100% (vs. 20.0%; *p* < 0.001), with no further improvement despite few-shot learning with 54 question–answer pairs.

When inaccurate outputs were reviewed for hallucinations, we found 112 (90.3%) fact-conflicting hallucinations (FCH) and 12 (9.7%) input-conflicting hallucinations (ICH) across all experiments. Hallucination type and distribution across each experiment are reported in Table 2. We did not find contextual-conflicting hallucinations (CCH) in any of our experiments.

**Table 2:** Hallucinations type and distribution across all experiments

Hallucination	Total	Fact-conflicting	Input-conflicting	Contextual-conflicting
BASELINE	57	48 (84.2%)	9 (15.8%)	-
Experiment 1	33	30 (90.9%)	3 (9.1%)	-
Experiment 2	22	22 (100%)	-	-
Experiment 3	10	10 (100%)	-	-
Experiment 4	1	1 (100%)	-	-
Experiment 5	1	1 (100%)	-	-

Interestingly, the two graders did not find any contextual-conflicting hallucination in any LLM-generated outputs.

### Text-similarity analysis

For the secondary outcomes, we found differences in the customized LLM framework compared to the baseline across similarity scores (BLEU score, ROUGE-LCS F1, METEOR Score F1, and our Custom OpenAI Score) for all questions (Table 3). The score average values for text-based and table-based questions, clinical scenarios, and graphical distributions of each score are reported in Supplementary Table 2 and Supplementary Fig. 1, respectively.

**Table 3:** Evaluation of text-to-text-similarity between LLM-generated outputs and human expert-provided answers used as the gold standard across all questions

Metrics	Baseline	Experiment 1	Experiment 2	Experiment 3	Experiment 4	Experiment 5
BLEU score:						
Mean (± SD)	0.025 (±0.023)	0.095 (±0.088)	0.111 (±0.143)	0.101 (±0.094)	0.140 (±0.119)	0.124 (±0.073)
Significance		*p* < 0.001	*p* < 0.001	*p* < 0.001	*p* < 0.001	*p* < 0.001
ROUGE-LCS F1:						
Mean (± SD)	0.201 (±0.053)	0.334 (±0.120)	0.347 (±0.138)	0.336 (±0.114)	0.345 (±0.119)	0.359 (±0.095)
Significance		*p* < 0.001	*p* < 0.001	*p* < 0.001	*p* < 0.001	*p* < 0.001
METEOR score F1:						
Mean (± SD)	0.308 (±0.059)	0.417 (±0.104)	0.429 (±0.126)	0.408 (±0.101)	0.428 (±0.115)	0.421 (±0.081)
Significance		*p* < 0.001	*p* < 0.001	*p* < 0.001	*p* < 0.001	*p* < 0.001
Custom OpenAI Score:						
Mean (± SD)	0.939 (±0.016)	0.954 (±0.017)	0.956 (±0.018)	0.956 (±0.016)	0.957 (±0.013)	0.958 (±0.017)
Significance		*p* < 0.001	*p* < 0.001	*p* < 0.001	*p* < 0.001	*p* < 0.001

Statistical testing is based on pairwise comparison (Mann–Whitney *U* Test) between each experimental setting and the baseline.

## Discussion

**Fig. 2:**
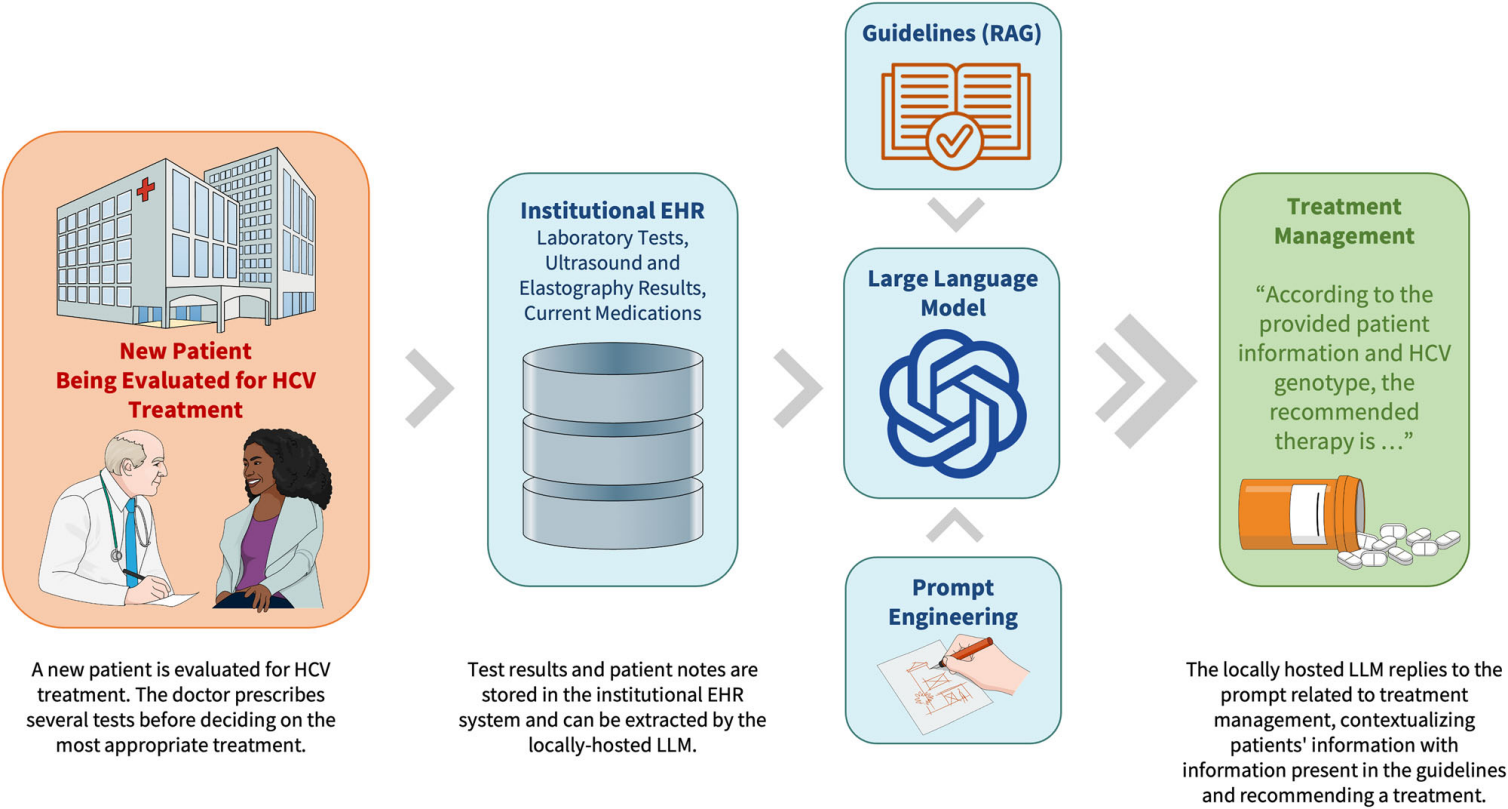
Example of a clinical decision support system integrated with large language models. When a patient is being evaluated for HCV treatment, the doctor prescribes several tests (laboratory and imaging), whose results are stored in the institutional EHR system. The locally hosted LLM has a standardized clinical scenario prompt with laboratory and imaging values that are directly extracted from EHR. Afterward, the standardized prompt is queried to the LLM, which has access to the relevant guidelines to recommend the most appropriate treatment. HCV Hepatitis C virus, EHR electronic health record, RAG retrieval augmented generation, LLM large language model.

Integrating LLMs into CDSSs may revolutionize healthcare delivery by leveraging natural language processing to interpret clinical documentation, aligning LLM-generated recommendations with current medical research and best practices^22,23^ (Fig. 2). For instance, a locally hosted LLM might be granted access to patient-specific data. This data can be integrated into a tailored prompt designed to identify the most appropriate treatment plan for a specific patient. The LLM, will have contemporary access to the guidelines and provide a recommendation on treatment based on guideline knowledge. However, before having LLM-aided CDSSs, it is necessary to define the best guidelines format that can maximize output accuracy.

We demonstrate the performance of our proposed framework in a subset of the potential questions that could be asked by physicians managing patients with chronic HCV. We identified an optimal framework for LLM-friendly clinical guidelines that achieves near-perfect accuracy and outperforms GPT-4 Turbo alone for answering questions about the management of HCV infection. The baseline GPT-4 Turbo showed an overall accuracy of 43.0%, consistent with other studies querying LLMs for management questions related to gastroenterology and hepatology, ranging from 25 to 90%^24–37^. This suggested that the model’s base knowledge was imperfect despite having access to information up to April 2023^38^.

Our findings also highlight the difficulty of LLMs to parse tables, with a clear improvement in performance after tables were converted to text-based lists, suggesting that information cannot be retrieved accurately from non-text sources. The difficulty of LLMs to parse tables is a known limitation^39^, and a critical technical issue that should be addressed since the medical literature often contains tables with important information for clinicians.

Modern LLMs such as GPT-4, according to their multimodal capabilities and context sensitivity, can interpret inputs from both images and textual elements^40^. OpenAI has noted that GPT-4 was tested on different benchmarks on textual, graphical, and visual elements (ChartQA^41^, AI2D^42^, DocVQA^43^, Infographic VQA^44^) with an accuracy range from 75.1% to 88.4% redefined the previously best models in these benchmarks which ranged from 61.2% to 88.4%^40^. Despite GPT-4 becoming state of the art in graphical-context interpretation, we demonstrate that it cannot interpret the non-text sources reported in the HCV guidelines, showing 16.0% overall accuracy in extracting pertinent information (as described in detail in Supplementary Note 1). Inaccuracies in graphical elements interpretation can result in the loss of critical information and context when converting non-text sources into a readable format for LLMs, which likely affected the GPT-4 Turbo’s ability to accurately interpret and reason with the information contained in non-text sources. This factor, coupled with the challenge of context retention across the segmented data in non-text sources, could have contributed to the lower performance in “reasoning and interpretation” tasks. These results imply that the information present in the guidelines should be presented as text (i.e., in LLM-friendly format) to be efficiently and accurately retrieved and interpreted by LLMs.

We found that the similarity scores (BLEU^45^, ROUGE-L^46^, METEOR^47^, and a custom OpenAI score) calculated between output generated by GPT-4 Turbo and the free text expert answers do not necessarily reflect differences in expert-graded qualitative accuracy. We found statistically significant differences across all similarity metrics when the outputs of the in-context guideline experiments were compared to the baseline outputs as reported in Table 3. Importantly, when we evaluated the in-context guideline experiments, we found no clear correlation with the change in similarity metrics with expert-graded qualitative accuracy. This has also been reported in other studies^18–21,48^ and may be explained by the fact that these scores were developed to measure word overlap, sentence structure similarity, and semantic coherence and not factual correctness. For clinical questions, factual correctness is the most important feature. This is an important challenge that should be addressed since current responses could appear lexically comparable to a reference answer but fail to capture the factual information necessary to guide clinical care. This can result in high scores for responses that are factually incorrect (false positives) or low scores for accurate responses that are phrased differently than the reference (false negatives). While useful for certain aspects of evaluation, these metrics fail to capture the nuances of medical relevance, completeness, and contextual correctness in the answers provided by the LLM. This limitation underscores the persistent need for expert physician oversight in the evaluation process (i.e., human-in-the-loop), with automated grading of LLM-generated responses still being an unresolved challenge.

We also found that few-shot learning did not improve performance above and beyond in-context learning, text formatting, table conversion, and prompt engineering (Fig. 1). This suggests that the model’s zero-shot querying capabilities were already robust without requiring few-shot strategies, which was previously described by reporting different one-shot vs. few-shot^49,50^.

Our work is limited by several factors. Firstly, we only investigate the application of LLM in the screening, diagnosis, and management of one disease across the spectrum of hepatology. However, our questions were representative of every section in the guideline, covering each major area of clinical management. Secondly, we ran each question for a limited number of iterations, with the performance being consistently excellent across multiple experiments. We do not vary the temperature setting for each of the questions and stages of the ablation study. We had limited resources to test the framework, though we acknowledge that the performance may differ with changes in the temperature parameter. Finally, we do not evaluate the performance of our framework with other LLMs, such as LlaMA^51^ or PaLM^52^. While these other models are used for many tasks, most studies using LLM in gastroenterology and hepatology have employed GTP 3.5 and GPT-4.0^24–37^.

In a recent study, Jin et al. developed LiVersa^53^, a liver disease-specific LLM using RAG and guidelines from the American Association for the Study of the Liver (AASLD), which showed notable limitations in providing completely accurate answers, especially in complex clinical scenarios. Also, from their methodology, it is unclear how they converted guidelines into text, the chunking strategy (which we do not employ in our framework), and their accuracy assessments and lack of data on output accuracy rates. Therefore, despite the similar aims, we cannot compare our study findings.

We present a novel LLM framework to generate answers to complex clinical questions with high accuracy, drawing from established guidelines for HCV management. We highlight the current limitations in LLM non-text sources interpretation and the benefit of in-context structured re-formatted guidelines with accompanied prompt engineering to guide understanding of the underlying text structure.

In conclusion, our results suggest that LLMs like GPT-4 Turbo are suitable for parsing clinical guidelines, but that their effectiveness can be enhanced by structured formatting strategies, prompt engineering, and text conversion of non-text sources. Moreover, our findings suggest that with appropriate reformatting, few-shot learning may not increase overall accuracy. We highlight the need for further research to enhance LLM’s ability to parse non-text sources and validate new metrics to evaluate not only similarity but also accuracy for clinical LLM applications.

## Methods

### Guidelines selection

We analyzed the current HCV guidelines from the prominent Northern American and European liver associations. Among these, we selected the European Association for the Study of the Liver (EASL) on the Hepatitis C Virus, entitled “EASL recommendations on treatment of hepatitis C: Final update of the series” published in 2020^54^, to explore our framework. The selected guideline comprised the most complex corpus of text containing broad recommendations on screening and management. In addition, the document contained in-depth information on drug-drug interactions, which was not reported in the Northern American^55,56^ guidelines. We also tested our framework on specific questions that were not addressed in the European guidelines using the most up-to-date Northern American HCV guidelines (as reported in Supplementary Note 3, Supplementary Table 3, Supplementary Table 4, Supplementary Fig. 3, Supplementary Fig. 4).

### Standardized prompts creation

Two expert hepatologists (M.G. and L.S.C.) drafted 20 representative questions (Table 4). Fifteen questions addressed screening and management recommendations from each of the major sections, including the guideline main text (10 questions) and graphical tables (5 questions). Tables are a standard feature of clinical guidelines and summarize recommendations in specific ways that may not be reflected in the text. In addition, the two experts drafted five comprehensive clinical cases, each reflecting different HCV-related management strategies, including best treatment selection, drug–drug interaction, and management of treatment severe adverse reactions. All the questions are structured to test reasoning and comprehension from both the main text and tables.

**Table 4:** List of questions

Text-based questions	
1.	A screening blood test before a knee replacement surgery revealed a positive HCV antibody—what test should be performed to confirm HCV infection?
2.	When a patient with HCV can be considered cured after HCV therapy?
3.	Is there any major contraindication to HCV therapy?
4.	Is it possible to apply treatment without determining genotype using grazoprevir/elbasvir?
5.	What are the recommended treatment regimens and duration for a patient with HCV genotype 3 and no cirrhosis?
6.	A patient on the transplantation list for HCC and decompensated liver cirrhosis should be treated before or after transplantation.
7.	Should patients with HCV-positive patients be listed for kidney transplant treated? If yes, why?
8.	Patients with fibrosis F3, according to elastography, should be continuing HCC screening after successful HCV eradication?
9.	Is HCV treatment during pregnancy recommended?
10.	When should children born by an HCV-positive mother be tested for HCV infection?
**Table-based questions**	
11.	What test can be used to assess the liver disease severity before treatment?
12.	Is there any interaction between cyclosporine and DAAs?
13.	Is there any interaction between apixaban and DAAs?
14.	Among anticoagulants and antiplatelets which is the one medication with the lowest risk of interactions with DAAs?
15.	What anticonvulsants are at higher risk of inducing drug interactions with DAAs?
**Clinical scenarios**	
16.	A 45-year-old male with an unremarkable medical history was scheduled for a routine inguinal hernia surgery. As part of the preoperative evaluation, he was tested for hepatitis C virus (HCV) antibodies, which returned positive. Subsequent HCV RNA testing confirmed active infection, and genotyping identified the virus as HCV genotype 1a. The patient had no prior knowledge of his HCV status and had never been tested or treated for hepatitis C. Before initiating treatment, a liver elastography was performed to assess liver health, yielding a liver stiffness measurement of 5 kPa. What is the recommended treatment for this patient (drugs and duration)?
17.	A 55-year-old patient, previously lost to follow-up, returns to the liver clinic with a history of failed interferon-based therapy for HCV genotype 3. Recent laboratory tests confirm active HCV infection with genotype 3, accompanied by elevated liver enzymes (AST: 100 IU/L, ALT: 150 IU/L). Additional laboratory results include bilirubin at 1.2 mg/dL, creatinine at 0.87 mg/dL, albumin at 3.9 g/dL, and an INR of 1.10. Liver elastography shows a liver stiffness measurement of 15 kPa, without clinical signs of liver decompensation, as observed in the physical examination. What is the recommended therapy for this patient?
18.	A 60-year-old patient with advanced chronic kidney disease (CKD) at stage 4 is diagnosed with Hepatitis C virus (HCV) infection. The patient’s current renal function parameters include a creatinine clearance of 28 mL/min. Additionally, the patient presents with decompensated liver cirrhosis, classified as Child-Pugh Class B8, indicating significant liver dysfunction. What is the recommended therapy for this patient?
19.	A 60-year-old female patient diagnosed with Hepatitis C virus (HCV) genotype 1a, who does not have liver cirrhosis, was recently prescribed a 12-week course of Sofosbuvir (400 mg)/Velpatasvir (100 mg). The patient has a significant medical history of atrial fibrillation, for which she is being treated with amiodarone. During the initial assessment with the hepatologist, the patient inadvertently omitted mentioning their amiodarone treatment. As of now, the patient has not commenced the HCV treatment. Is it advisable for the patient to promptly inform her hepatologist about the amiodarone treatment before starting the HCV therapy?
20.	A 70-year-old female with a recent diagnosis of Hepatitis C Virus (HCV) genotype 1a, confirmed to have no evidence of liver cirrhosis, commenced a treatment regimen consisting of a 12-week course of Sofosbuvir (400 mg) combined with Velpatasvir (100 mg) daily. The patient’s baseline liver function tests were within normal limits, with an Alanine Aminotransferase (ALT) level of 45 IU/L (normal range: 30–45 IU/L). However, upon re-evaluation 4 weeks post-treatment initiation, her ALT levels had markedly elevated to 1123 IU/L. Should the prescribed HCV treatment be discontinued in light of this significant ALT elevation?

Two expert hepatologists drafted 20 questions that specifically refer to information about management recommendations addressing information contained in the guideline main text (10 questions), graphical tables (5 questions), and clinical scenarios (5 questions).

### Ablation study: customized LLM framework

We used a combination of RAG using EASL HCV guidelines, in different experimental settings with increasing degrees of complexity regarding guideline reformatting, prompt architecture, and few-shot learning to create a customized framework applied to the GPT-4 Turbo model (released by OpenAI, in November 2023 with knowledge updated until April 2023^38^). Experiments with the OpenAI’s Application Programming Interface (API) *v. 1.17* cannot directly retrieve information from .*pdf* files. Therefore, the original pdf guidelines document was converted to a .*txt* file with UTF-8 encoding using the Python (*v. 3.11*) library PyPDF2 v3.0.

**Fig. 3:**
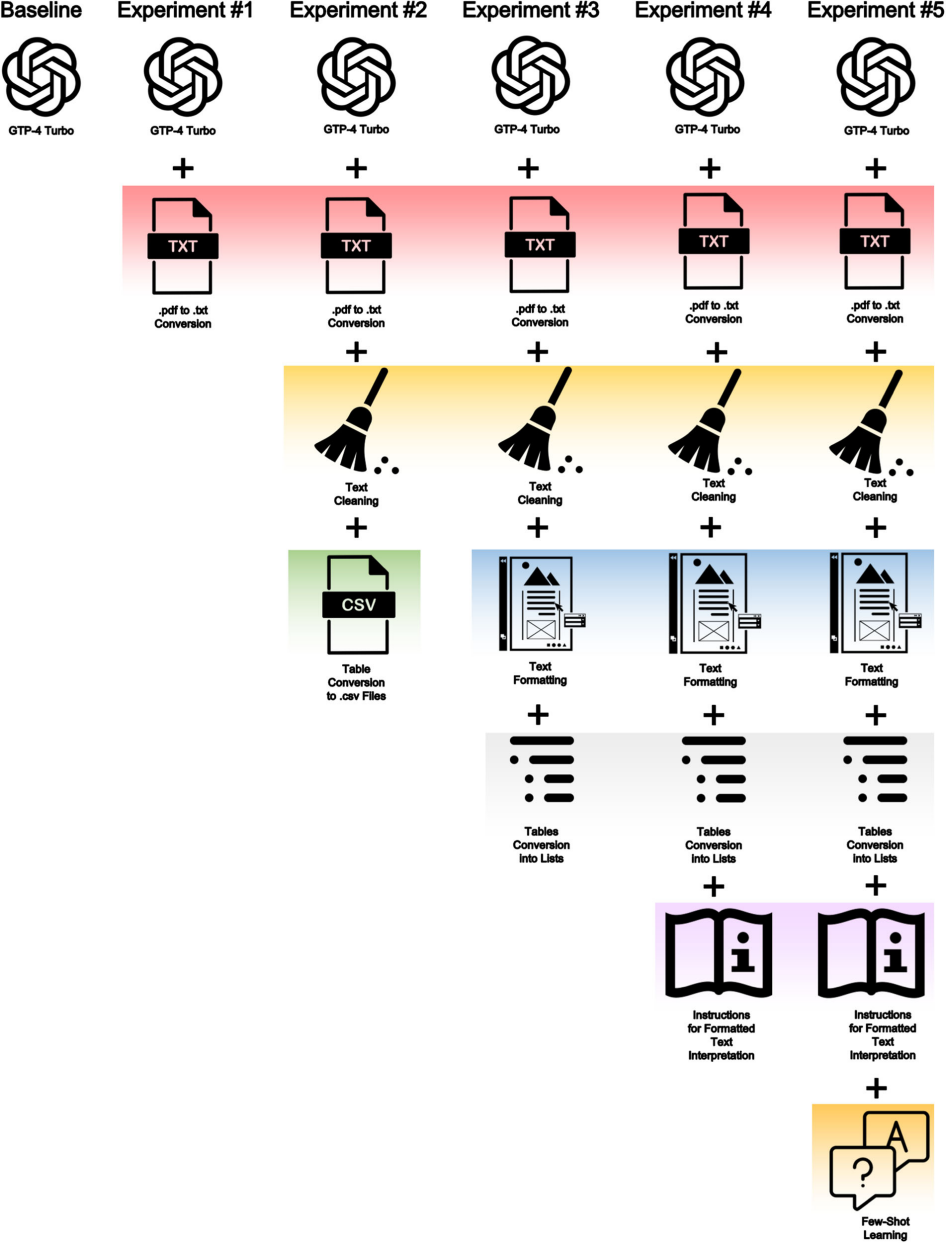
Depiction of Ablation Study experimental settings (Experiment 1 through Experiment 5) to investigate how guideline reformatting, prompt architecture, and few-shot learning impact the accuracy and robustness of LLM outputs.

We carried out an ablation study from the baseline (Experiments 1 through 5) to investigate how different settings in guideline reformatting, prompt architecture, and few-shot learning impact the accuracy and robustness of LLM outputs (Fig. 3). It is still unknown how non-text sources (e.g., graphical tables and flowcharts) are processed by LLMs and whether the information extracted is accurate. Therefore, we performed preliminary experiments to test the accuracy of the GPT image conversion process (Supplementary Note 1) and found very low accuracy (16.0%) in extracting pertinent table information, with accuracy ranging from 0% (graphical tables) to 48.0% (only text tables). In light of these findings, we introduced text conversion of tables (non-text sources) into text-based lists and tested their impact on accuracy in Experiments 3, 4, and 5.

#### Baseline

Use of the foundational GPT-4 Turbo without any context. For this experiment, we only provided the questions without any further instruction.

#### Experiment 1

Use of the foundational GPT-4 Turbo with guidelines uploaded in context after pdf-to-text conversion in UTF-8 encoding without any additional text cleaning processes.

#### Experiment 2

Use of the foundational GPT-4 Turbo with guidelines uploaded in context after being manually cleaned with the removal of non-informative data (e.g. page header and bibliography). Tables presented as images in the original text were manually converted into *.csv* files and then provided as context.

#### Experiment 3

Use of the foundational GPT-4 Turbo with guidelines uploaded as context that were cleaned and formatted to provide a consistent structure alongside the whole document. In addition, we converted all tables from *.csv* files into text-based lists and included them in the main text. Each paragraph title was preceded by “Paragraph Title”. All the paragraph recommendations were collected and organized into a list preceded by “Paragraph Recommendations”. Evidence reported in the main text was organized and preceded by “Paragraph Text”.

#### Experiment 4

Use of the foundational GPT-4 Turbo with guidelines uploaded as context that were cleaned and formatted, with tables converted into text-based lists. We also provided a series of prompts (i.e., prompt engineering) that instructed the model on how to interpret the structured guidelines (Supplementary Table 1).

#### Experiment 5

Use of the foundational GPT-4 Turbo with guidelines uploaded as context that were cleaned and formatted, with tables converted into text-based lists. We included the series of prompts (i.e., prompt engineering) and added a series of 54 question-answer pairs (i.e. few-shot learning) (Supplementary Table 1).

The experiments are summarized in Fig. 3 and were conducted on a local Python environment with OpenAI API access. Instructions, when provided, are summarized in Supplementary Table 1. We used foundational model default parameters, selecting a temperature of 0.9, and setting a maximum number of tokens in output equivalent to 800.

### Primary outcome

Our primary outcome was to evaluate qualitative rates of accuracy according to expert grading based on the information reported in EASL guidelines^54^. We repeated the query 5 times each for the 20 questions for each experimental setting and reported the proportion of accurate responses. Each answer was graded with a score of 1 if the text contained completely accurate information or 0 otherwise. Two expert hepatologists (M.G., with four years of experience in treating HCV patients, and L.S.C., with thirty years of experience in treating HCV patients) manually graded each response. The two graders were blind to each other and towards the experimental setting when labeling answers. Disagreements in grading occurred for 5.0% of outputs and were solved by consensus between the two graders.

When outputs are considered inaccurate, the inaccuracy is caused by hallucinations (i.e., the production of plausible sounding but potentially unverified or incorrect information)^57,58^. According to the recent definitions of Zhang et al., we defined three types of hallucinations: FCH, ICH, and CCH^59^.

### Secondary outcome

Our secondary outcome was to evaluate the similarity of LLM-generated responses to the human expert-provided answers used as the gold standard. In particular, an expert hepatologist (M.G.) provided a single answer for each of the 20 questions, which was reviewed and approved by the second expert hepatologist (L.S.C.), and then used as the gold standard expert response to which LLM responses were compared in text-similarity using Recall-Oriented Understudy for Gisting Evaluation (ROUGE)^46^, Bilingual Evaluation Understudy (BLEU)^45^, Metric for Evaluation of Translation with Explicit Ordering (METEOR)^47^, and a Custom OpenAI score (for in-depth explanation see Supplementary Note 2). The Custom OpenAI score is based on cosine similarity, while the other scores are based on word overlap and semantic coherence between two text sources. We evaluated the similarity by comparing LLM-generated answers to the corresponding ones provided by experts. All these scores are expressed on a scale from 0 to 1, where a score of 1 denotes perfect alignment between two compared text sources. The mean and standard deviation of the similarities were estimated after repeating the query 5 times each for the 20 questions.

### Statistical analysis

We employed the Chi-Square Test to compare accuracy among experiments qualitatively. We employed the Mann-Whitney U Test to compare differences among continuous scoring for automatic evaluation of answers. We considered statistically significant a two-tailed *p*-value < 0.05. To conduct the analysis, we used Python *v 3.11* and SciPy *v 1.11*.

### Supplementary information

Supplementary Information

## Data Availability

All LLMs prompts are included in the Summary Information with the prompts used. For any additional information, please contact the corresponding authors.

## Code availability

Code can be provided based on personal requests, please contact the corresponding authors.

## References

[1] Peng C (2023). A study of generative large language model for medical research and healthcare. NPJ Digit. Med..

[2] Thirunavukarasu AJ (2023). Large language models in medicine. Nat. Med..

[3] Meskó B (2023). The imperative for regulatory oversight of large language models (or generative AI) in healthcare. NPJ Digit. Med..

[4] Singhal K (2023). Large language models encode clinical knowledge. Nature.

[5] Webster P (2023). Six ways large language models are changing healthcare. Nat. Med..

[6] Nagulu I (2023). Clinical guidelines and best practices. Glob. J. Res. Anal..

[7] Mignini, L. Review of clinical practice guidelines. In *Systematic Reviews to Support Evidence-Based Medicine* 165–170 (CRC Press, Boca Raton, 2022). 10.1201/9781003220039-15.

[8] Liu Y (2023). Summary of ChatGPT-Related research and perspective towards the future of large language models. Meta-Radiol..

[9] Mesko B (2023). The ChatGPT (Generative Artificial Intelligence) revolution has made artificial intelligence approachable for medical professionals. J. Med. Internet Res..

[10] Nori, H. et al. Capabilities of GPT-4 on medical challenge problems. *arxiv* https://arxiv.org/abs/2303.13375 (2023).

[11] Scheurer, J. et al. Technical report: large language models can strategically deceive their users when put under pressure. arxiv https://arxiv.org/abs/2311.07590 (2023).

[12] Lewis, P. et al. Retrieval-augmented generation for knowledge-intensive NLP tasks. *arxiv* https://arxiv.org/abs/2005.11401 (2020).

[13] Ouyang, L. et al. Training language models to follow instructions with human feedback. *arxiv* https://arxiv.org/abs/2203.02155 (2022).

[14] Falade-Nwulia O (2017). Oral direct-acting agent therapy for hepatitis C virus infection. Ann. Intern. Med..

[15] Moore JD (2023). Physician-level determinants of HCV screening during pregnancy in a U.S. sample. Arch. Gynecol. Obstet..

[16] Southern WN (2014). Physician nonadherence with a hepatitis C screening program. Qual. Manag; Health Care.

[17] Elimination of hepatitis by 2030. https://www.who.int/health-topics/hepatitis/elimination-of-hepatitis-by-2030#tab=tab_1.

[18] Chen, A. et al. Evaluating Question Answering Evaluation. In *Proc. 2nd Workshop on Machine Reading for Question Answering* 119–124 (Association for Computational Linguistics, Stroudsburg, PA, USA, 2019). 10.18653/v1/D19-5817.

[19] Tang L (2023). Evaluating large language models on medical evidence summarization. NPJ Digit. Med..

[20] Blagec, K. et al. A global analysis of metrics used for measuring performance in natural language processing. In *Proc. NLP Power! The First Workshop on Efficient Benchmarking in NLP* 52–63 (Association for Computational Linguistics, Stroudsburg, PA, USA, 2022). 10.18653/v1/2022.nlppower-1.6.

[21] Fabbri AR (2021). SummEval: re-evaluating summarization evaluation. Trans. Assoc. Comput Linguist.

[22] Mahadevaiah G (2020). Artificial intelligence‐based clinical decision support in modern medical physics: Selection, acceptance, commissioning, and quality assurance. Med. Phys..

[23] Golden, G. et al. Applying artificial intelligence to clinical decision support in mental health: what have we learned? *Health Policy Technol,* 100844 10.1016/j.hlpt.2024.100844 (2024).

[24] Tariq R (2024). Evolving landscape of large language models: an evaluation of ChatGPT and bard in answering patient queries on colonoscopy. Gastroenterology.

[25] Lahat A (2023). Evaluating the utility of a large language model in answering common patients’ gastrointestinal health-related questions: are we there yet?. Diagnostics.

[26] Lee T-C (2023). ChatGPT answers common patient questions about colonoscopy. Gastroenterology.

[27] Gorelik Y (2023). language models for streamlined postcolonoscopy patient management: a novel approach. Gastrointest. Endosc..

[28] Henson JB (2023). Evaluation of the potential utility of an artificial intelligence chatbot in gastroesophageal reflux disease management. Am. J. Gastroenterol..

[29] Emile SH (2023). How appropriate are answers of online chat-based artificial intelligence (ChatGPT) to common questions on colon cancer?. Surgery.

[30] Moazzam Z (2023). Quality of ChatGPT responses to questions related to pancreatic cancer and its surgical care. Ann. Surg. Oncol..

[31] Cankurtaran, R. E. et al. Reliability and usefulness of ChatGPT for inflammatory bowel diseases: an analysis for patients and healthcare professionals. *Cureus*10.7759/cureus.46736 (2023). 10.7759/cureus.46736 PMC10630704 38022227

[32] Levartovsky A (2023). Towards AI-augmented clinical decision-making: an examination of ChatGPT’s utility in acute ulcerative colitis presentations. Am. J. Gastroenterol..

[33] Patil NS (2023). Using artificial intelligence chatbots as a radiologic decision-making tool for liver imaging: do chatgpt and bard communicate information consistent with the ACR appropriateness criteria?. J. Am. Coll. Radiol..

[34] Pugliese, N. et al. Accuracy, reliability, and comprehensibility of chatgpt-generated medical responses for patients with nonalcoholic fatty liver disease. *Clin. Gastroenterol. Hepatol.*10.1016/j.cgh.2023.08.033 (2023). 10.1016/j.cgh.2023.08.033 37716618

[35] Endo Y (2023). Quality of ChatGPT responses to questions related to liver transplantation. J. Gastrointest. Surg..

[36] Cao JJ (2023). Accuracy of information provided by ChatGPT regarding liver cancer surveillance and diagnosis. Am. J. Roentgenol..

[37] Yeo YH (2023). Assessing the performance of ChatGPT in answering questions regarding cirrhosis and hepatocellular carcinoma. Clin. Mol. Hepatol..

[38] OpenAI. New models and developer products announced at DevDay. https://openai.com/blog/new-models-and-developer-products-announced-at-devday.

[39] Sui, Y. et al. Table meets LLM: can large language models understand structured table data? A benchmark and empirical study. *arxiv* https://arxiv.org/abs/2305.13062 (2023).

[40] OpenAI et al. GPT-4 technical report. https://arxiv.org/abs/2303.08774 (2023).

[41] Masry, A. et al. ChartQA: a benchmark for question answering about charts with visual and logical reasoning. *arxiv* https://arxiv.org/abs/2203.10244 (2022).

[42] Kembhavi, A. et al. A diagram is worth a dozen images. in 235–251. 10.1007/978-3-319-46493-0_15 (2016).

[43] Mathew, M. et al. DocVQA: a dataset for VQA on document images. *arxiv* https://arxiv.org/abs/2007.00398 (2020).

[44] Mathew, M. et al. InfographicVQA. In *2022 IEEE/CVF Winter Conference on Applications of Computer Vision (WACV)* 2582–2591 (IEEE, 2022). 10.1109/WACV51458.2022.00264.

[45] Papineni, K. et al. BLEU: a method for automatic evaluation of machine translation. In *Proc*. *40th Annual Meeting of the Association for Computational Linguistics* 311–318 (Association of Computational Machinery, 2002).

[46] Lin, C.-Y. Rouge: a package for automatic evaluation of summaries. *In:* Text summarization branches, 74–82 (2004).

[47] Banerjee, S. et al. METEOR: an automatic metric for MT evaluation with improved correlation with human judgments. In *Proc. ACL Workshop on Intrinsic and Extrinsic Evaluation Measures for Machine Translation and/or Summarization*. 65–72 (2005).

[48] Zhang, T. et al. BERTScore: evaluating text generation with BERT. *arxiv* https://arxiv.org/abs/1904.09675 (2019).

[49] Agrawal, M. et al. Large language models are few-shot clinical information extractors. *arxiv* https://arxiv.org/abs/2205.12689 (2022).

[50] Hu, Y. et al. Improving large language models for clinical named entity recognition via prompt engineering. *arxiv* https://arxiv.org/abs/2303.16416 (2023). 10.1093/jamia/ocad259 PMC11339492 38281112

[51] Touvron, H. et al. Llama 2: open foundation and fine-tuned chat models. *arxiv* https://arxiv.org/abs/2307.09288 (2023).

[52] Anil, R. et al. PaLM 2 Technical Report. *arxiv* https://arxiv.org/abs/2305.10403 (2023).

[53] Ge J. et al. Development of a liver disease-specific large language model chat interface using retrieval augmented generation. 10.1101/2023.11.10.23298364 (2023). 10.1097/HEP.0000000000000834 PMC11706764 38451962

[54] Pawlotsky J-M (2020). EASL recommendations on treatment of hepatitis C: final update of the series✰. J. Hepatol..

[55] Bhattacharya, D. et al. Hepatitis C guidance 2023 update: american association for the study of liver diseases– infectious diseases society of america recommendations for testing, managing, and treating hepatitis c virus infection. *Clin. Infect. Dis.*10.1093/cid/ciad319 (2023). 10.1093/cid/ciad319 37229695

[56] Ghany MG (2020). Hepatitis C guidance 2019 update: american association for the study of liver diseases–infectious diseases society of america recommendations for testing, managing, and treating hepatitis C virus infection. Hepatology.

[57] Giuffrè, M. et al. L. Evaluating ChatGPT in medical contexts: the imperative to guard against hallucinations and partial accuracies. *Clin. Gastroenterol. Hepatol.*10.1016/j.cgh.2023.09.035 (2023). 10.1016/j.cgh.2023.09.035 37863408

[58] Giuffrè, M. et al. Scrutinizing ChatGPT Applications in gastroenterology: a call for methodological rigor to define accuracy and preserve privacy. *Clin. Gastroenterol. Hepatol.*10.1016/j.cgh.2024.01.024 (2024). 10.1016/j.cgh.2024.01.024 38311148

[59] Zhang, Y. et al. Siren’s song in the ai ocean: a survey on hallucination in large language models. *arxiv* https://arxiv.org/abs/2309.01219 (2023).

