# Supplementary material for: Optimization of hepatological clinical guidelines interpretation by large language models: a retrieval augmented generation-based framework
Source: NPJ Digit Med. 2024 Apr 23;7:102. doi: 10.1038/s41746-024-01091-y (PMC11039454; doi:10.1038/s41746-024-01091-y)
Supplement: Supplementary file 1 — Supplementary Information [file 41746_2024_1091_MOESM1_ESM.docx]

**Supplementary Information**

**Supplementary Note 1: Digital Image Processing (Non-Text Sources)**

We employed GPT-4 to extract relevant information from Tables. GPT-4 is an LLM with multimodal competence to analyze both text and complex images. To address GPT-4 accuracy in extracting relevant information from guidelines’ non-text sources, we extracted the 16 tables reported in the guideline as images (*.png* format). Then, we defined the following prompt: “*You are given an image representing a table that may contain information reported as text or graph in the form of symbols embedded in a colored background. Extract all the relevant information and report it as a structured text, ensuring that all the data are accurately represented in the same order as it appears in the original image*”. After the prompt was entered, each image was provided as an external file. The same prompt request was entered separately 10 times for each image ensuring that each iteration was independently entered from the previous one. The experiments were conducted on a local Python environment with OpenAI API access. Each response was assessed by two grades (S.K. and M.G.) and was defined as accurate if the extracted text reported all the information present in the original source, maintaining the same order and structure.

**Supplementary Note 2: Quantitative Analysis of Text Similarity**

**ROUGE (Recall-Oriented Understudy for Gisting Evaluation).** ROUGE is a set of metrics for evaluating automatic summarization and machine translation systems. It works by comparing an automatically produced summary or translation with a set of reference summaries or translations (usually human-generated). There are several variations of the ROUGE metric, including ROUGE-N: Measures n-gram overlap between the generated text and reference text. For example, ROUGE-1 refers to the overlap of 1-gram (each word), while ROUGE-2 refers to the overlap of bi-grams (two consecutive words). ROUGE-L: Uses the longest common subsequence (LCS) between the generated text and the reference text. It considers sentence-level structure similarity and identifies the longest co-occurring in-sequence n-grams. ROUGE-U: This is less commonly used and refers to unigram-based metrics without stop word removal. These metrics mainly assess the presence and frequency of matching chunks of text, with ROUGE-L also considering the order of the text^48^.

**BLEU (Bilingual Evaluation Understudy).** BLEU is a metric for evaluating a generated text against one or more reference texts. BLEU measures the precision of the generated text, which is the percentage of n-grams in the generated text that appear in the reference text. It applies a brevity penalty to prevent overly short generations from scoring too high^47^.

**METEOR (Metric for Evaluation of Translation with Explicit Ordering).** METEOR is another metric for evaluating translated text against a human-produced reference translation. It is based on the harmonic mean of unigram precision and recall, with recall weighted higher than precision. It also considers synonyms and stemming, and has a mechanism to align words and phrases that are semantically equivalent but not exact matches. Unlike BLEU, it aims to address some of the inadequacies by incorporating a more nuanced matching process^49^.

**Customized OpenAI Score.** We developed a metric called OpenAI score that is a metric for evaluating the similarity between two sentences. The similarity score is determined by calculating the cosine similarity to evaluate semantic alignment between two vectorized sentences which represent the distance in an embedding space. The sentences are vectorized in the “text-embedding-ada-002” embedding space provided by OpenAI^40^.

| Table 1. Complete Prompt-Engineering report for all experiments. |
| --- |
| Prompt-Engineering |
| Baseline |
| No prompt was provided, with the exception of the clinical question. |
| Experiment 1 |
| This is a context-based interaction. Based on the following context {Guidelines Text}, answer the following question: {Query}. |
| Experiment 2 |
| This is a context-based interaction. Based on the following context {Guidelines Text} and {Tables .csv Files}, answer the following question: {Query}. |
| Experiment 3 |
| This is a context-based interaction. Based on the following context {Guidelines Text*}, answer the following question: {Query}.  **Tables were converted into lists.* |
| Experiment 4 |
| This is a context-based interaction. Based on the following context {Guidelines Text} that has been formatted according to the following {Instructions*}, answer the following question: {Query}.  **Instructions:*  These are a series of instructions on how to interpret the text structure:   - Paragraph Title: Recognize this as the main topic or the section header. It gives context to the information that follows. - Paragraph Recommendations: you will find bullet-point directives or guidelines that are meant to be actionable items. Each bullet point is a discrete recommendation with a grading or priority level indicated by the letter and number in parentheses (e.g., A1), which is crucial for understanding the recommendation's weight. - Paragraph Text: This provides the evidence for what is included in the paragraph recommendation.   Besides, you will find in the Text references to Tables that are reported alongside the text and reported as lists. |
| Experiment 5 |
| This is a context-based interaction. Based on the following context {Guidelines Text} that has been formatted according to the following {Instructions*} and a series of {Question-Answer Pairs**} that are examples on how you should provide answers, answer the following question: {Query}.  **Instructions:*  These are a series of instructions on how to interpret the text structure:   - Paragraph Title: Recognize this as the main topic or the section header. It gives context to the information that follows. - Paragraph Recommendations: you will find bullet-point directives or guidelines that are meant to be actionable items. Each bullet point is a discrete recommendation with a grading or priority level indicated by the letter and number in parentheses (e.g., A1), which is crucial for understanding the recommendation's weight. - Paragraph Text: This provides the evidence for what is included in the paragraph recommendation.   Besides, you will find in the Text references to Tables that are reported alongside the text and reported as lists.  **Question-Answer Pairs:  {"Q": "What are the available direct-acting antiviral (DAA) drug combinations for treating hepatitis C in Europe?",  "A": "The available direct-acting antiviral (DAA) drug combinations for treating hepatitis C in Europe include the following: 1) Sofosbuvir: Available as tablets containing 400 mg of sofosbuvir for once-daily intake, or in half-strength tablets containing 200 mg of sofosbuvir for once-daily intake ; 2) Sofosbuvir/Velpatasvir: This combination is available in tablets containing 400 mg of sofosbuvir and 100 mg of velpatasvir, taken once daily. There are also half-strength tablets containing 200 mg of sofosbuvir and 50 mg of velpatasvir, as well as granules containing 50 mg of sofosbuvir and 12.5 mg of velpatasvir, taken once daily according to body weight ; 3) Sofosbuvir/Velpatasvir/Voxilaprevir: This is a combination tablet containing 400 mg of sofosbuvir, 100 mg of velpatasvir, and 100 mg of voxilaprevir, taken once daily with food ; 4) Glecaprevir/Pibrentasvir: Available in tablets containing 100 mg of glecaprevir and 40 mg of pibrentasvir, taken three tablets once daily with food. Alternatively, it's available in film-coated granules in sachets containing 50 mg of glecaprevir and 20 mg of pibrentasvir, mixed in a small amount of food and taken three to five sachets once daily according to body weight ; 5) Grazoprevir/Elbasvir: This combination is available as tablets containing 100 mg of grazoprevir and 50 mg of elbasvir, taken once daily."},  {"Q": "How should a patient with suspected newly acquired HCV infection be initially tested?", "A": "For patients with suspected newly acquired Hepatitis C Virus (HCV) infection, the following initial testing is recommended: 1) Testing for Anti-HCV Antibodies: All patients should be tested for anti-HCV antibodies; 2) Testing for HCV RNA or HCV Core Antigen: Along with anti-HCV antibodies, either HCV RNA or HCV core antigen should be tested in serum or plasma. /n If a patient tests positive for anti-HCV antibodies but negative for HCV RNA or HCV core antigen, they should be retested for HCV RNA 12 and 24 weeks later to confirm definitive clearance of the virus."},  {"Q": "What follow-up testing is recommended for patients with suspected de novo HCV infection who initially test negative for HCV RNA or HCV core antigen?", "A": "For patients suspected of having a de novo recently acquired HCV infection who initially test negative for HCV RNA or HCV core antigen, it is recommended to conduct a follow-up test for HCV RNA or HCV core antigen at 12 and 24 weeks after the initial negative result. This retesting is crucial to confirm the definitive clearance of the virus, ensuring that the initial negative test accurately reflects the absence of an HCV infection."},  {"Q": "What is the first-line diagnostic test for patients with suspected chronic HCV infection?", "A": "The first-line diagnostic test for patients with suspected chronic Hepatitis C Virus (HCV) infection is the testing for anti-HCV antibodies in serum or plasma. If anti-HCV antibodies are detected in these patients, further testing should be carried out to determine either HCV RNA or HCV core antigen."},  {"Q": "How should HCV reinfection be suspected and confirmed?", "A": "Hepatitis C Virus (HCV) reinfection should be suspected and confirmed based on the following criteria: 1) Suspecting Reinfection: Reinfection should be suspected if there is a detection of HCV-RNA more than 12 or 24 weeks post-Sustained Virologic Response (SVR), especially in individuals who continue to engage in risk behaviors for HCV infection; 2) Confirming Reinfection: The diagnosis of reinfection is confirmed by the reappearance of HCV RNA or HCV core antigen after achieving SVR. Additionally, genetic sequencing followed by phylogenetic analysis is used to demonstrate that the infection is caused by a different genotype or a distantly related strain of the same genotype from the initial infection; 3) Monitoring for Reinfection: It is recommended that HCV RNA-negative or HCV core antigen-negative individuals be retested for HCV RNA or HCV core antigen 12 and 24 weeks after a negative result to confirm definitive clearance of the virus. This helps identify any cases of reinfection that may occur after spontaneous or treatment-induced clearance of HCV, particularly among those with ongoing risk factors for infection."},  {"Q": "What is the recommended method for determining anti-HCV antibodies in serum or plasma?", "A": "The recommended method for determining anti-HCV (Hepatitis C Virus) antibodies in serum or plasma is by Enzyme Immunoassay (EIA). This method is part of the screening strategy for HCV infection and should ideally align with the local epidemiology of HCV infection within the context of local, regional, or national action plans. Additionally, whole blood sampled on dried blood spots (DBSs) can be used as an alternative to serum or plasma obtained by venipuncture for anti-HCV antibody testing. These samples are typically sent to a central laboratory where the EIA will be performed. Furthermore, rapid diagnostic tests (RDTs) using various matrices such as serum, plasma, fingerstick whole blood, or oral fluid (crevicular fluid/saliva) can be utilized as point-of-care tests to facilitate anti-HCV antibody screening and improve access to care."},  {"Q": "What is the lower limit of detection for HCV RNA by a sensitive molecular method?", "A": "The lower limit of detection for HCV RNA by a sensitive molecular method is ≤ 15 IU/ml. This level of sensitivity is recommended for the diagnosis of both recently acquired and chronic Hepatitis C Virus (HCV) infection. In settings where such sensitive HCV RNA assays are not available or affordable, alternative methods with a lower sensitivity threshold are considered, such as point-of-care assays with a lower limit of detection ≤ 1000 IU/ml (3.0 Log10 IU/ml)."},  {"Q": "Can HCV core antigen be used as an alternative to HCV RNA for diagnosing HCV viraemia?", "A": "Yes, HCV core antigen can be used as an alternative to HCV RNA for diagnosing HCV viraemia. HCV core antigen in serum or plasma is a marker of HCV replication. Its detection is applicable for diagnosing viraemia in cases of recently acquired HCV infection, chronic infection, or after HCV reinfection. However, it's important to note that HCV core antigen assays are generally less sensitive than HCV RNA assays."},  {"Q": "What alternative HCV RNA assay can be used when sensitive assays are not available or affordable?", "A": "When sensitive HCV RNA assays are not available or affordable, an alternative is to use a low-cost point-of-care test for HCV RNA detection. This alternative assay should have a lower limit of detection ≤ 1000 IU/ml (3.0 Log10 IU/ml). This approach allows for broader access to HCV diagnosis and care in settings where high-sensitivity assays may be impractical due to cost or availability constraints."},  {"Q": "What is the primary goal of HCV therapy?", "A": "The primary goal of Hepatitis C Virus (HCV) therapy is to cure the infection, and this objective is pursued with several key aims: 1) Preventing Complications: The cure of HCV infection is vital to prevent the complications associated with HCV-related liver and extrahepatic diseases. This includes preventing hepatic necroinflammation, fibrosis, cirrhosis, decompensation of cirrhosis, hepatocellular carcinoma (HCC), severe extrahepatic manifestations, and death; 2) Improving Quality of Life and Removing Stigma: Successful therapy aims to improve the overall quality of life for patients and help in removing the social stigma associated with HCV infection; 3) Preventing Onward Transmission of HCV: Treating HCV infection is also a method of preventing the onward transmission of the virus, an approach known as treatment as prevention (TasP)."},  {"Q": "What constitutes the endpoint of HCV therapy?", "A": "The endpoint of Hepatitis C Virus (HCV) therapy is defined as follows: 1) Undetectable HCV RNA: The primary endpoint is achieving undetectable HCV RNA in serum or plasma. This should be determined by an assay with a lower limit of detection ≤ 15 IU/ml; 2) Timeframe for Assessment: The assessment for undetectable HCV RNA should be done 12 weeks (SVR12) or 24 weeks (SVR24) after the end of treatment."},  {"Q": "Can HCV core antigen levels be used as an alternative endpoint of therapy?", "A": "Yes, HCV core antigen levels can be used as an alternative endpoint of therapy for Hepatitis C Virus (HCV) infection. The standard endpoint of HCV therapy is undetectable HCV RNA in serum or plasma, as assessed by an assay with a lower limit of detection ≤ 15 IU/ml, 12 weeks (SVR12) or 24 weeks (SVR24) after the end of treatment. However, undetectable HCV core antigen in serum or plasma at these same time points (SVR12 or SVR24) can be used as an alternative endpoint in patients who had detectable HCV core antigen prior to therapy."},  {"Q": "What surveillance is recommended for patients with advanced fibrosis or cirrhosis after achieving SVR?", "A": "For patients with advanced fibrosis (F3) or cirrhosis (F4) who have achieved a Sustained Virologic Response (SVR) after Hepatitis C Virus (HCV) treatment, the following surveillance recommendations are made: 1) Hepatocellular Carcinoma (HCC) Surveillance: These patients should undergo surveillance for HCC every 6 months using ultrasound. This is because, although the risk of developing de novo or incident HCC is reduced following SVR, it is not completely eliminated; 2) Surveillance for Esophageal Varices: In patients with cirrhosis, surveillance for esophageal varices should be performed by endoscopy. This is recommended if varices were present at pre-treatment endoscopy or if certain clinical parameters are met, such as a platelet count falling below 150,000 and an increase in elastography to more than 20 kPa; 3) Monitoring for HCV Reinfection: The risk of HCV reinfection should be addressed, especially in at-risk populations. Regular monitoring for HCV reinfection, through bi-annual or at least annual HCV RNA assessments, should be conducted in populations with ongoing risk behavior, such as people who inject drugs (PWIDs) or men who have sex with men (MSM)."},  {"Q": "Who should be offered treatment for HCV infection?", "A": "Treatment for Hepatitis C Virus (HCV) infection should be offered to the following groups: 1) All Treatment-Naïve and Treatment-Experienced Patients: This includes those with recently acquired or chronic HCV infection. They must be offered treatment without delay; 2) Patients with Significant Fibrosis or Cirrhosis: This specifically refers to those with a METAVIR score of F2, F3, or F4. It includes both compensated (Child-Pugh A) and decompensated (Child-Pugh B or C) cirrhosis. Urgent treatment should be considered for these patients; 3) Patients with Clinically Significant Extrahepatic Manifestations: Treatment should also be prioritized for patients experiencing significant health issues related to HCV that affect organs or systems outside the liver."},  {"Q": "Why might treatment not be recommended for patients with limited life expectancy due to non-liver-related comorbidities?", "A": "Treatment for Hepatitis C Virus (HCV) might not be recommended for patients with limited life expectancy due to non-liver-related comorbidities for several reasons: 1) Cost-Benefit Analysis: In patients with a significantly limited life expectancy due to other serious health conditions, the benefits of HCV treatment might not outweigh the costs or potential risks associated with the therapy; 2) Treatment Priority: The primary goal of HCV treatment is to improve liver health and prevent long-term complications like cirrhosis or hepatocellular carcinoma. In patients with limited life expectancy from other conditions, the urgency and potential benefit of treating HCV diminish; 3) Resource Allocation: In settings with limited healthcare resources, prioritizing HCV treatment for patients who are likely to have a longer-term benefit from the therapy might be considered more effective; 4) Quality of Life Considerations: For patients with severe non-liver-related comorbidities, the focus of care may be more on palliative measures and maintaining quality of life rather than on aggressive treatments for conditions like HCV. /n This recommendation is graded B2, indicating a moderate level of evidence and consensus among experts."},  {"Q": "What pre-therapeutic assessment should be conducted prior to initiating HCV therapy?", "A": "The pre-therapeutic assessment prior to initiating Hepatitis C Virus (HCV) therapy should include the following key elements: 1) Proof of HCV Viremia: This involves confirming the presence of HCV infection, which can be done by detecting HCV RNA or HCV core antigen in the patient's blood; 2) Determination of the Presence or Absence of Cirrhosis: Assessing whether cirrhosis is present is crucial, and this should be done using a non-invasive method (such as liver elastography). The presence of cirrhosis can influence the choice of treatment regimen and the need for post-treatment surveillance; 3) The patient should be screened for concomitant HBV/HIV infection and a full liver function test (LFTs) blood panel and renal function test (creatinine) should be carried out; 4) Drug-Drug Interaction Check: It is important to carefully assess for any possible drug-drug interactions between the HCV treatment and other medications the patient may be taking. This includes implementing necessary dose modifications if required. /n These steps are essential to ensure that the selected HCV treatment regimen is both safe and effective for the patient."},  {"Q": "How should liver disease severity be assessed before HCV treatment?", "A": "Using non-invasive tests based on laboratory test (e.g., APRI) or liver stiffness” "},  {"Q": "Why is it important to identify cirrhosis before starting HCV therapy?", "A": "Identifying cirrhosis before starting Hepatitis C Virus (HCV) therapy is crucial for several reasons:1) Determining Treatment Duration: The presence of advanced fibrosis (F3) or cirrhosis (F4) influences the duration of treatment with certain HCV genotypes and regimens. This determination is essential to optimize the treatment plan for each patient.; 2) Surveillance for Hepatocellular Carcinoma (HCC): Patients with cirrhosis need post-treatment surveillance for HCC if treatment for HCC is available. Identifying cirrhosis allows for the implementation of appropriate monitoring strategies to manage this risk.; 3) Treatment Efficacy: In certain cases, such as patients infected with HCV genotype 3 and compensated cirrhosis (Child-Pugh A), lower sustained virologic response rates (SVR12) may be observed. Knowing the stage of liver disease helps in anticipating these challenges and planning for potential retreatment strategies if needed. /n Non-invasive marker scores like FIB-4 or APRI can be used to assess the presence of advanced fibrosis or cirrhosis. In cases where this information is not available, a universal treatment duration of 12 weeks is recommended, regardless of the regimen used"},  {"Q": "What non-invasive methods should be used to assess liver fibrosis stage?", "A": "For the assessment of liver fibrosis stage in chronic Hepatitis C, non-invasive methods are recommended prior to therapy, as they can effectively evaluate liver disease severity. The following non-invasive methods are commonly used: 1) Liver Stiffness Measurement: This method is used to assess liver fibrosis and the presence of portal hypertension in patients with chronic Hepatitis C. It provides a non-invasive way to determine the extent of liver damage. ; 2) Assessment of Cirrhosis and Advanced Fibrosis: Diagnosing clinically inapparent cirrhosis (METAVIR score F4) or advanced (bridging) fibrosis (METAVIR score F3) is essential, as the choice of treatment regimen and post-treatment prognosis, including surveillance for hepatocellular carcinoma (HCC) every 6 months, depend on the stage of fibrosis. ; 3) Portal Hypertension: In patients with cirrhosis, non-invasive methods are also used to assess for portal hypertension, including the potential presence of esophageal varices. /n It's important to note, however, that non-invasive methods should not be used to assess fibrosis stage after therapy, as they are considered unreliable in this context"},  {"Q": "What factors may affect liver stiffness measurement?", "A": "Liver stiffness measurement, a non-invasive method used in assessing liver fibrosis, can be affected by several factors:1) Obesity: Excess body weight can interfere with the accuracy of liver stiffness measurements.; 2) High ALT Levels: Elevated levels of alanine aminotransferase (ALT), a liver enzyme, can influence the measurement results.; 3) Post-Prandial Testing: Performing liver stiffness measurements after eating (post-prandial) may also affect the accuracy of the results."},  {"Q": "What is the importance of assessing drug-drug interactions before starting HCV therapy?", "A": "The importance of assessing drug-drug interactions before starting Hepatitis C Virus (HCV) therapy lies in ensuring the safety and efficacy of the treatment. Here are the key reasons: 1) Safety: Many medications, including over-the-counter and prescribed drugs, can interact with HCV Direct-Acting Antivirals (DAAs). These interactions can lead to adverse effects, reduced effectiveness of the HCV therapy, or toxicity.; 2) Efficacy: Assessing interactions helps in optimizing the efficacy of HCV treatment. Certain drugs can interfere with the metabolism of DAAs, potentially diminishing their therapeutic effect.; 3) Personalized Treatment: Every patient may have a unique medication profile. A thorough assessment allows for tailoring the HCV treatment to individual needs, considering other medications the patient is taking.; 4) Avoiding Contraindications: Some drugs are contraindicated with certain HCV therapies. Identifying these beforehand prevents the use of harmful combinations.; 5) Compliance and Adherence: Understanding potential interactions aids in planning a treatment regimen that minimizes inconvenience and maximizes adherence to therapy. ; 6) Regular Updates and Changes in Medications: Medication profiles may change over time. Assessing drug-drug interactions at the start sets a precedent for continuous monitoring throughout the course of treatment. /n The guidelines emphasize that a thorough drug-drug interaction risk assessment is required before starting HCV therapy, based on the prescribing information for each DAA."},  {"Q": "How should drug-drug interactions be managed in HIV-HCV coinfected patients?", "A": "Managing drug-drug interactions in HIV-HCV coinfected patients involves several key steps; 1) Thorough Risk Assessment: Before initiating HCV therapy and when starting other medications during treatment, a thorough drug-drug interaction risk assessment is required. This assessment should be based on the prescribing information for each Direct-Acting Antiviral (DAA).; 2) Attention to Antiretroviral Drugs: Close attention must be paid to antiretroviral drugs used in the treatment of HIV. This includes identifying those that are contraindicated, not recommended, or require dose adjustment when used with particular DAA regimens.; 3) Utilizing Resources for Drug Interactions: Healthcare providers are advised to refer to summary data on key interactions, which can be found in the EASL guidelines document. "},  {"Q": "What should patients be educated about regarding adherence to HCV therapy and drug interactions?", "A": "Patients undergoing Hepatitis C Virus (HCV) therapy should be educated on several key aspects to ensure effective treatment and safety: 1) Adherence to Therapy: Patients should be informed about the critical importance of adhering to their therapy. This includes following the prescribed dosing recommendations precisely.; 2) Reporting Medication Use: Patients need to report the use of any other medications they are taking. This encompasses prescribed medications, over-the-counter medications, medications acquired via the internet, and the use of party or recreational drugs.; 3) Drug-Drug Interactions: Awareness of potential drug-drug interactions is essential. Patients should be informed about the possibility of interactions between their HCV therapy and other drugs they may be taking, and the potential need for dosage adjustments or the avoidance of certain drugs.; 4) Monitoring and Communication: Regular monitoring and open communication with healthcare providers are important. Patients should be encouraged to report any side effects or concerns and to maintain regular appointments for monitoring treatment efficacy and safety. /n These recommendations are graded A1, indicating a high level of importance and reliability based on evidence."},  {"Q": "Are there any interactions between HCV DAAs and nucleoside reverse transcriptase inhibitors (NRTIs)?", "A": "For interactions between Hepatitis C Virus (HCV) Direct-Acting Antivirals (DAAs) and Nucleoside Reverse Transcriptase Inhibitors (NRTIs), the following information is provided: 1) Abacavir, Emtricitabine, Lamivudine: No interaction is expected with Sofosbuvir (SOF), Ledipasvir/Sofosbuvir (SOF/VEL), Voxilaprevir/Velpatasvir/Sofosbuvir (SOF/VEL/VOX), Glecaprevir/Pibrentasvir (GLE/PIB), or Grazoprevir/Elbasvir (GZR/EBR).; 2) Tenofovir Disoproxil Fumarate (TDF): No interaction is expected with Sofosbuvir (SOF). However, there may be potential interactions with Ledipasvir/Sofosbuvir (SOF/VEL) and Voxilaprevir/Velpatasvir/Sofosbuvir (SOF/VEL/VOX) that could require dosage adjustment. No interaction is expected with Glecaprevir/Pibrentasvir (GLE/PIB) and Grazoprevir/Elbasvir (GZR/EBR). ; 3) Tenofovir Alafenamide (TAF): No interaction is expected with Sofosbuvir (SOF), Ledipasvir/Sofosbuvir (SOF/VEL), Voxilaprevir/Velpatasvir/Sofosbuvir (SOF/VEL/VOX), Glecaprevir/Pibrentasvir (GLE/PIB), or Grazoprevir/Elbasvir (GZR/EBR)."},  {"Q": "What are the interactions between HCV DAAs and non-nucleoside reverse transcriptase inhibitors (NNRTIs)?", "A": "The interactions between HCV Direct-Acting Antivirals (DAAs) and non-nucleoside reverse transcriptase inhibitors (NNRTIs) vary based on the specific drugs involved. For instance:* Doravirine: There is no interaction expected with any of the listed DAAs.; * Efavirenz: While there is no interaction expected with sofosbuvir (SOF) alone, efavirenz should not be co-administered with combinations of SOF/VEL (sofosbuvir/velpatasvir), SOF/VEL/VOX (sofosbuvir/velpatasvir/voxilaprevir), GLE/PIB (glecaprevir/pibrentasvir), or GZR/EBR (grazoprevir/elbasvir) due to potential adverse interactions. /n These interactions highlight the importance of considering potential drug-drug interactions when prescribing HCV treatment regimens, particularly in patients who are on NNRTIs for HIV treatment."},  {"Q": "How do protease inhibitors interact with HCV DAAs?", "A": "Protease inhibitors interact with HCV Direct-Acting Antivirals (DAAs) in several ways: 1) Impact on Drug Levels: Protease inhibitors like grazoprevir and elbasvir are substrates of the cytochrome P450 3A (CYP3A) enzyme and P-glycoprotein (P-gp). Inducers of these proteins, such as efavirenz, etravirine, phenytoin, carbamazepine, bosentan, modafinil, and St John’s wort, can cause a marked decrease in plasma exposure to both DAAs and are therefore contraindicated. Strong inhibitors of CYP3A, which may significantly increase plasma concentrations of these DAAs, are either contraindicated or not recommended.; 2) Drug-Drug Interactions: There may be potential interactions with other DAAs like sofosbuvir/velpatasvir (SOF/VEL) and grazoprevir/elbasvir (GZR/EBR) which may require dosage adjustments. Sofosbuvir/velpatasvir/voxilaprevir (SOF/VEL/VOX) and glecaprevir/pibrentasvir (GLE/PIB) should not be co-administered with protease inhibitors.; 3) Contraindications in Decompensated Cirrhosis: Protease inhibitors are contraindicated in patients with decompensated cirrhosis. For example, glecaprevir/pibrentasvir should not be used in patients with decompensated cirrhosis post-liver transplantation. /n The interactions between protease inhibitors and DAAs can have significant implications on the efficacy and safety of HCV treatment, necessitating careful consideration and monitoring during therapy."},  {"Q": "What is the cutoff value for FibroScan® when diagnosing cirrhosis (F4) in HCV-positive patients? And what is the grade of confidence of this value?", "A": "The cutoff value for diagnosing cirrhosis (F4) in HCV-positive patients using FibroScan® is 13 kPa. The grade of confidence for this value, as indicated by the AUROC (Area Under the Receiver Operating Characteristic curve), ranges between 0.90 and 0.93, which reflects a high level of accuracy in the diagnostic performance of FibroScan® for cirrhosis in this patient group. For additional information, please refer to 'European Association for the Study of the Liver (EASL) recommendations on treatment of hepatitis C: Final update of the series' (DOI: https://doi.org/10.1016/j.jhep.2020.08.018)."},  {"Q": "How should HCV treatment be approached in pregnant women?", "A": "The approach to HCV treatment in pregnant women involves careful consideration due to the lack of extensive safety and efficacy data. The general recommendation is that HCV treatment during pregnancy is not advised in the absence of conclusive safety and efficacy data. However, treatment can be considered on a case-by-case basis for pregnant women or in cases of accidental conception during treatment. This decision should be made after a thorough discussion with the patient about the potential risks and benefits and should involve a coordinated approach with both hepatology and obstetric services. Additionally, breastfeeding is not contraindicated in women with HCV, except in cases of bleeding or cracked nipples, where specialist advice should be sought."},  {"Q": "What is the recommended treatment for HCV in patients with renal impairment?", "A": "The recommended treatment for Hepatitis C (HCV) in patients with renal impairment, including those on hemodialysis, involves specific considerations. For patients with severe renal impairment (eGFR <30 ml/min/1.73 m2) and those with end-stage renal disease on hemodialysis, the treatment should be carried out in expert centers with close monitoring by a multidisciplinary team. The general recommendations for HCV DAAs (Direct-Acting Antivirals) apply to these patients, with no need for dose adjustments. The preferred choices for patients with severe renal impairment and those requiring hemodialysis are the fixed-dose combination of glecaprevir and pibrentasvir and, for patients infected with HCV genotype 1b, the fixed-dose of grazoprevir and elbasvir. In patients with decompensated cirrhosis and mild to moderate renal impairment, treatment should be guided by their specific health status and liver function."},  {"Q": "What are the recommended DAAs for treating HCV genotype 1?", "A": "For treating HCV genotype 1, the recommended Direct-Acting Antivirals (DAAs) include: 1. Sofosbuvir/Velpatasvir, with an SVR12 observed in 98% of patients with genotype 1a and 99% with genotype 1b; 2. Glecaprevir/Pibrentasvir, effective for patients with compensated cirrhosis, showing SVR12 rates of 98%% for genotype 1a and 100%% for genotype 1b; 3. Grazoprevir/Elbasvir for genotype 1b, especially in those with severe renal impairment or on hemodialysis."},  {"Q": "What are the specific considerations for treating HCV in children and adolescents?", "A": "For treating Hepatitis C (HCV) in children and adolescents, the following considerations are important: 1) Testing: All children born to HCV-infected women should be tested for HCV infection from the age of 18 months. ; 2) Treatment Regimens: For adolescents aged 12-17 years who are treatment-naïve or treatment-experienced, without cirrhosis or with compensated (Child-Pugh A) cirrhosis, treatment should be according to general recommendations in adult patients. This includes using the fixed-dose combination of sofosbuvir (400 mg) and velpatasvir (100 mg) in a single tablet administered once daily, or the fixed-dose combination of glecaprevir (300 mg) and pibrentasvir (120 mg) in 3 tablets (100 mg/40 mg) administered once daily with food. ; 3) Clinical Trials and Safety Profile: * The DORA clinical trial, conducted in children and adolescents infected with HCV genotype 1, 2, 3, or 4, used the fixed-dose combination of glecaprevir and pibrentasvir for 8 to 16 weeks. In this study, all 47 adolescents aged 12-17 years achieved SVR12 (sustained virological response 12 weeks after treatment) with a safety profile and exposure consistent with that in adults. Pharmacokinetic exposures of glecaprevir and pibrentasvir were comparable to those in adults. /n * The most common adverse events observed in the trial were headache, fatigue, and nausea in adolescents; vomiting, cough, and headache in 6- to 11-year-old children; and vomiting in 3- to 5-year-old children. /n These considerations ensure that the treatment of HCV in children and adolescents is safe, effective, and based on evidence from clinical trials specific to this age group."},  {"Q": "How should HCV treatment regimens be adjusted in patients with decompensated cirrhosis?", "A": "In patients with decompensated cirrhosis, HCV treatment regimens need to be adjusted considering the following: 1) Avoidance of Certain DAAs: Direct-Acting Antivirals (DAAs) that include protease inhibitors such as grazoprevir, glecaprevir, or voxilaprevir are contraindicated in decompensated cirrhosis due to the risk of further liver decompensation and significant side effects. ; 2) Selection of Safe Regimens: The treatment should focus on DAA regimens that are safe for use in patients with decompensated cirrhosis. This usually involves the use of DAAs that do not have significant hepatic metabolism or are not associated with hepatotoxicity. ; 3) Dose Adjustments: Depending on the severity of liver dysfunction, dose adjustments of certain medications might be required to avoid accumulation and potential toxicity. ; 4) Monitoring for Adverse Effects: Close monitoring for adverse effects and signs of worsening liver function is essential, as these patients are at a higher risk of complications. ; 5) Managing Complications: Treatment of HCV in the context of decompensated cirrhosis should be coordinated with management of cirrhosis-related complications like ascites, variceal bleeding, and hepatic encephalopathy. ; 6) Consideration for Transplantation: In some cases, liver transplantation might be considered, and the timing of antiviral therapy in relation to transplantation needs careful planning. /n It's important to tailor the HCV treatment approach to the individual patient's needs and the specific characteristics of their liver disease."},  {"Q": "What are the considerations for HCV therapy in patients with compensated and decompensated cirrhosis?", "A": "For HCV therapy in patients with compensated and decompensated cirrhosis, the following considerations are important: 1) Compensated Cirrhosis (Child-Pugh A): Patients with compensated cirrhosis generally require careful selection of DAA regimens, considering the potential for more advanced liver disease. These patients can be treated effectively with most DAAs, but monitoring for side effects and liver function is important.; 2) Decompensated Cirrhosis (Child-Pugh B or C): In patients with decompensated cirrhosis, the choice of DAA regimen needs to be more cautious. Some DAA regimens, particularly those containing protease inhibitors (like grazoprevir, glecaprevir, or voxilaprevir), are contraindicated due to the risk of worsening liver function and potential side effects. Treatment should focus on regimens that are safe and effective in this context, with close monitoring for any signs of decompensation. ; 3) Drug-Drug Interactions: Given that patients with cirrhosis often take other medications, assessing potential drug-drug interactions is crucial, especially with medications used to manage complications of cirrhosis. ; 4) Monitoring: Regular monitoring of liver function tests, renal function, and other relevant parameters is essential during treatment, as patients with cirrhosis have a higher risk of adverse events. ; 5) Post-Treatment Surveillance: Even after achieving SVR, patients with cirrhosis, particularly decompensated cirrhosis, require ongoing surveillance for hepatocellular carcinoma (HCC) and other complications of cirrhosis."},  {"Q": "How is the sustained virological response (SVR) measured in HCV therapy?", "A": "The sustained virological response (SVR) in HCV therapy is measured by the absence of detectable HCV RNA in a patient's serum or plasma. This is typically assessed by an assay with a lower limit of detection ≤ 15 IU/ml. SVR is usually evaluated 12 weeks (SVR12) or 24 weeks (SVR24) after the end of treatment. Achieving SVR is considered a cure of HCV infection. In some cases, undetectable HCV core antigen in serum or plasma after the end of treatment can also be used as an alternative measure of SVR, especially in patients with detectable HCV core antigen prior to therapy."},  {"Q": "Why are cytochrome P450/P-gp-inducing agents contraindicated with DAA regimens?", "A": "Cytochrome P450/P-gp-inducing agents are contraindicated with Direct-Acting Antiviral (DAA) regimens because they can significantly reduce the concentrations of HCV DAAs in the body. These agents, such as carbamazepine, phenytoin, and phenobarbital, induce the activity of enzymes that metabolize drugs in the liver, leading to increased clearance and decreased effectiveness of DAAs. This reduction in drug levels can compromise the efficacy of the HCV treatment, potentially leading to treatment failure and the development of viral resistance. Therefore, it's important to avoid these inducers or switch to alternative medications that do not interact with DAAs."},  {"Q": "How should patients with a history of liver transplantation and HCV be managed?", "A": "Patients with a history of liver transplantation and HCV should be managed with prompt and effective antiviral treatment. These patients are considered a high-priority group for treatment due to the risk of rapid progression of liver disease and graft loss. The choice of treatment regimen should be tailored based on the HCV genotype, the presence of cirrhosis, renal function, and potential drug-drug interactions, particularly with immunosuppressive agents. Close monitoring of liver function, HCV RNA levels, and potential side effects of the treatment is also essential in this patient population. The goal is to achieve sustained virological response (SVR), thereby improving graft survival and overall patient outcomes."},  {"Q": "What are the recommendations for HCV testing in low-incidence populations?", "A": "In low-incidence populations, the recommendations for HCV testing include replacing anti-HCV antibody screening with low-cost point-of-care tests for viraemia with a lower limit of detection ≤ 1000 IU/ml (3.0 Log10 IU/ml) or for HCV core antigen testing, when such tests are available. This approach is recommended if it is less costly than anti-HCV antibody testing. This strategy is particularly suggested for populations where the prevalence of HCV is low, as it allows for a more efficient allocation of resources while ensuring that those infected with HCV are identified and can be directed towards appropriate care and treatment."},  {"Q": "What are the guidelines for reflex testing in HCV diagnosis?", "A": "The guidelines for reflex testing in HCV diagnosis recommend that if anti-HCV antibodies are detected, the presence of HCV RNA by molecular assay or HCV core antigen by EIA in serum or plasma should be determined to identify patients with active viraemia. This process, known as reflex testing, is essential for confirming an active HCV infection. It enables a more efficient diagnostic pathway by immediately following up an antibody-positive result with a test for viral replication, thus shortening the time to diagnosis and treatment. Reflex testing is particularly important because the presence of anti-HCV antibodies alone does not distinguish between past and current infections."},  {"Q": "Why is it important to confirm viraemia in HCV patients before initiating treatment?", "A": "Confirming viraemia in HCV patients before initiating treatment is crucial because it indicates active infection. The presence of HCV RNA or HCV core antigen in serum or plasma confirms ongoing viral replication, which is a key criterion for starting antiviral therapy. Without evidence of viraemia, treatment may not be necessary, as the presence of anti-HCV antibodies alone could indicate a past infection that has already resolved. Therefore, confirming active viral replication ensures that treatment is targeted to those patients who will benefit from it, thereby optimizing healthcare resources and avoiding unnecessary treatment."},  {"Q": "Can Glecaprevir/Pibrentasvir be administered in granule form?", "A": "Yes, Glecaprevir/Pibrentasvir can be administered in granule form. The granules are film-coated and come in sachets containing 50 mg of Glecaprevir and 20 mg of Pibrentasvir. These granules should be mixed together in a small amount of food and taken once daily. The dosage may vary between three to five sachets daily, depending on the patient's body weight. This form of administration offers an alternative to tablets and can be particularly useful for patients who have difficulty swallowing pills."},  {"Q": "What education should patients receive regarding adherence to HCV therapy?", "A": "Patients undergoing HCV therapy should receive education emphasizing the importance of adherence to the prescribed treatment regimen. They should be informed about following the dosing recommendations precisely and the significance of consistent medication intake for the success of the treatment. Additionally, patients should be educated about reporting any use of other prescribed medications, over-the-counter medications, medications bought via the internet, and the use of party or recreational drugs. This education is critical as it can significantly affect the efficacy of the treatment and help in managing potential drug-drug interactions."},  {"Q": "What are the guidelines for reflex testing in HCV diagnosis?", "A": "The guidelines for reflex testing in HCV diagnosis include the following: If anti-HCV antibodies are detected, it is recommended to perform reflex testing for HCV RNA or HCV core antigen in serum or plasma. This approach helps to identify patients with active viraemia. Reflex testing is crucial as it shortens the pathway to care by providing a prompt diagnosis and facilitating the initiation of treatment if needed. It streamlines the diagnostic process, ensuring that those who test positive for HCV antibodies are quickly assessed for active infection."},  {"Q": "When should liver biopsy be considered in HCV patients?", "A": "Liver biopsy should be considered in HCV patients in cases where there is uncertainty in the diagnosis or potential additional etiologies of liver disease that cannot be clarified through non-invasive methods. It is reserved for situations where non-invasive assessments are inconclusive or when additional information about the liver condition is needed that cannot be obtained through other means."},  {"Q": "What non-invasive methods are recommended for assessing fibrosis stage in HCV patients?", "A": "For assessing the fibrosis stage in HCV patients, non-invasive methods are recommended, including liver stiffness measurement and serum biomarkers. Inexpensive and reliable biomarker panels like APRI (Aspartate Aminotransferase to Platelet Ratio Index) and FIB-4 (Fibrosis-4) are used for this purpose. These non-invasive methods provide a practical and less risky alternative to liver biopsy for evaluating the extent of liver fibrosis in patients with HCV."},  {"Q": "What additional infections should be tested for in HCV patients before starting therapy?", "A": "Before starting therapy, HCV patients should be tested for past or current Hepatitis B Virus (HBV) infection, for HIV infection, and for immunity to Hepatitis A Virus (HAV). Testing for these additional infections is crucial because co-infections can influence the choice of HCV treatment regimen, affect the prognosis, and require specific management strategies."},  {"Q": "How should alcohol consumption and substance abuse be addressed in HCV patients?", "A": "In HCV patients, alcohol consumption and substance abuse should be carefully assessed and quantified. Specific counseling should be provided to these patients as part of their pre-therapeutic assessment. Addressing alcohol consumption and substance abuse is crucial, as these factors can contribute to the progression of liver disease and impact the effectiveness of HCV treatment."},  {"Q": "What are some contraindications to current DAA-based treatments?", "A": "The contraindications to current Direct-Acting Antiviral (DAA)-based treatments include: 1) The use of certain cytochrome P450/P-glycoprotein-inducing agents such as carbamazepine, phenytoin, and phenobarbital. These agents are contraindicated with all HCV DAA regimens if they cannot be switched to other medications due to the risk of significantly reduced concentrations of HCV DAAs. 2) Treatment regimens that comprise an HCV protease inhibitor, such as grazoprevir, glecaprevir, or voxilaprevir, are contraindicated in patients with decompensated (Child-Pugh B or C) cirrhosis and in patients with previous episodes of decompensation."},  {"Q": "What defines the endpoint of HCV therapy?", "A": "The endpoint of HCV therapy is defined as undetectable HCV RNA in serum or plasma by an assay with a lower limit of detection ≤ 15 IU/ml 12 weeks (SVR12) or 24 weeks (SVR24) after the end of treatment. Alternatively, undetectable HCV core antigen in serum or plasma 12 weeks (SVR12) or 24 weeks (SVR24) after the end of treatment can be used as an endpoint in patients with detectable HCV core antigen prior to therapy. Additionally, using a qualitative HCV RNA assay with a lower limit of detection ≤ 1000 IU/ml (3.0 Log10 IU/ml) is acceptable as an alternative endpoint where sensitive HCV RNA assays are not available or affordable."},  {"Q": "What are the main objectives of curing HCV infection?", "A": "The main objectives of curing HCV infection are to: 1) prevent the complications of HCV-related liver and extrahepatic diseases including hepatic necroinflammation, fibrosis, cirrhosis, decompensation of cirrhosis, hepatocellular carcinoma (HCC), severe extrahepatic manifestations, and death; 2) improve quality of life and remove the stigma associated with the disease; and 3) prevent onward transmission of HCV, adopting the approach of 'treatment as prevention'."},  {"Q": "Is there any drug interaction between DAAs and carbamazepine?", "A": "Yes, there are significant drug interactions between carbamazepine and direct-acting antiviral agents (DAAs) used for treating Hepatitis C Virus (HCV) infection. Carbamazepine is a cytochrome P450 and P-glycoprotein (P-gp) inducing agent. Its use is contraindicated with all HCV DAA regimens due to the risk of significantly reduced concentrations of HCV DAAs, which could lead to a high risk of virological failure. Specifically, carbamazepine should not be co-administered with the following DAAs: Sofosbuvir (SOF), Sofosbuvir/Velpatasvir (SOF/VEL), Sofosbuvir/Velpatasvir/Voxilaprevir (SOF/VEL/VOX), Glecaprevir/Pibrentasvir (GLE/PIB), and Grazoprevir/Elbasvir (GZR/EBR)."},  {"Q": "Is there any interaction between warfarin and DAAs?", "A": "Yes, there are potential interactions between warfarin and various direct-acting antiviral agents (DAAs) used in the treatment of Hepatitis C Virus (HCV) infection. These interactions may require dosage adjustments or additional monitoring. Specifically, warfarin has potential interactions with the following DAAs:   - Sofosbuvir (SOF) - Sofosbuvir/Velpatasvir (SOF/VEL) - Sofosbuvir/Velpatasvir/Voxilaprevir (SOF/VEL/VOX) - Glecaprevir/Pibrentasvir (GLE/PIB) - Grazoprevir/Elbasvir (GZR/EBR)."},   {"Q": "Is there any interaction between bisoprolol and DAAs?", "A": " There is no interaction expected between bisoprolol, a beta-blocker commonly used for treating hypertension, and any direct-acting antiviral agents (DAAs). This indicates that you can continue using bisoprolol safely while undergoing treatment with DAAs for HCV infection. However, it's always best to inform your healthcare provider about all the medications you are taking, to ensure the most effective and safe treatment plan."},  {"Q": " My doctor prescribed sovaldi to treat HCV infection. However, I forgot to mention that I am currently taking atorvastatin. Should I call the doctor office back before starting therapy?", "A": "There is no clinically significant interaction expected between atorvastatin and Sofosbuvir (brand name: Sovaldi), which is the DAA you have been prescribed for HCV treatment. This means you can safely continue taking atorvastatin while undergoing treatment with Sovaldi. However, it is always a good practice to inform your healthcare provider about all the medications you are taking, including atorvastatin, to ensure the safest and most effective treatment plan."},  {"Q": " I am currently taking abilify to treat my bipola disorder. However, I was prescribed sovaldi to treat my current HCV infection. Is there any interaction between the two medications?", "A": " There is no specific information in my knowledge source about the interaction between Abilify (aripiprazole) and Sovaldi (sofosbuvir), the direct-acting antiviral agent you've been prescribed for HCV treatment. However, it is crucial to inform your healthcare provider about all medications you are taking, including Abilify, to ensure the safest and most effective treatment plan."},  {"Q": "I am worried that the medication that I take to treat my hypertension (amlodipine) may interfere with mavyret. Do you now if there is any interaction?", "A": " There is no interaction expected between amlodipine, a medication commonly used to treat hypertension, and any direct-acting antiviral agents (DAAs), including Mavyret (glecaprevir/pibrentasvir). This means you can safely continue taking amlodipine while undergoing treatment with Mavyret for your HCV infection. However, it is always advisable to inform your healthcare provider about all the medications you are taking to ensure the safest and most effective treatment plan."} |

| Table 2. Evaluation of text-to-text similarity between LLM-generated outputs and human-expert provided answers used as the gold-standard. This table reports the metrics for questions categorized by their referral to text, tables, or clinical scenarios. | | | | | | |
| --- | --- | --- | --- | --- | --- | --- |
| Metrics | Baseline | Experiment 1 | Experiment 2 | Experiment 3 | Experiment 4 | Experiment 5 |
| Text-based Questions | | | | | | |
| BLEU score |  |  |  |  |  |  |
| Mean (± SD) | .033 (± .028) | .139 (± .097) | .157 (± .188) | .140 (± .113) | .194 (± .142) | .153 (± .088) |
| Significance |  | p < 0.001 | p < 0.001 | p < 0.001 | p < 0.001 | p < 0.0001 |
| ROUGE-LCS F1 |  |  |  |  |  |  |
| Mean (± SD) | .225 (± .057) | .411 (± .108) | .407 (± .164) | .399 (± .120) | .403 (± .131) | .405 ± .102) |
| Significance |  | p < 0.0001 | p < 0.001 | p < 0.001 | p < 0.001 | p < 0.001 |
| METEOR score F1 |  |  |  |  |  |  |
| Mean (± SD) | .329 (± .059) | .466 (± .119) | .448 (± .154) | .439 (± .125) | .453 (± .147) | .451 ± .094) |
| Significance |  | p < 0.001 | p < 0.001 | p < 0.001 | p < 0.001 | p < 0. 001 |
| Custom OpenAI Score |  |  |  |  |  |  |
| Mean (± SD) | .944 (± .016) | .961 (± .016) | .960 (± .020) | .960 (± .018) | .958 (± .014) | . 961 ± .020) |
| Significance |  | p < 0.001 | p < 0.001 | p < 0.001 | p < 0.001 | p < 0.001 |
| Table-based Questions | | | | | | |
| BLEU score |  |  |  |  |  |  |
| Mean (± SD) | .014 (± .014) | .033 (± .037) | .057 (± .048) | .056 (± .048) | .077 (± .067) | .095 (± .044) |
| Significance |  | p = 0.077 | p < 0.001 | p < 0.001 | p < 0.001 | p < 0.001 |
| ROUGE-LCS F1 |  |  |  |  |  |  |
| Mean (± SD) | .180 (± .037) | .243 (± .060) | .287 (± .066) | .271 (± .073) | .272 (± .084) | .323 (± .069) |
| Significance |  | p < 0.001 | p < 0.001 | p < 0.001 | p < 0.001 | p < 0.001 |
| METEOR score F1 |  |  |  |  |  |  |
| Mean (± SD) | .315 (± .042) | .364 (± .058) | .413 (± .105) | .363 (± .063) | .390 (± .073) | .366 (± .048) |
| Significance |  | p = 0.001 | p < 0.001 | p = 0.003 | p < 0.001 | p < 0.001 |
| Custom OpenAI Score |  |  |  |  |  |  |
| Mean (± SD) | .940 (± .009) | .952 (± .015) | .958 (± .012) | .954 (± .014) | .956 (± .011) | .958 (± .013) |
| Significance |  | p < 0.001 | p < 0.001 | p < 0.001 | p < 0.001 | p < 0.001 |
| Clinical Scenarios | | | | | | |
| BLEU score |  |  |  |  |  |  |
| Mean (± SD) | .021 (± .016) | .067 (± .046) | .073 (± .033) | .070 (± .039) | .094 (± .029) | .093 (± .029) |
| Significance |  | p < 0.001 | p < 0.001 | p < 0.001 | p < 0.001 | p < 0.001 |
| ROUGE-LCS F1 |  |  |  |  |  |  |
| Mean (± SD) | .173 (± .031) | .270 (± .075) | .286 (± .061) | .278 (± .054) | .302 (± .044) | .301 (± .043) |
| Significance |  | p < 0.001 | p < 0.001 | p < 0.001 | p < 0.001 | p < 0.001 |
| METEOR score F1 |  |  |  |  |  |  |
| Mean (± SD) | .263 (± .051) | .373 (± .045) | .407 (± .069) | .394 (± .045) | .418 (± .042) | .417 (± .041) |
| Significance |  | p < 0.001 | p < 0.001 | p < 0.001 | p < 0.001 | p < 0.001 |
| Custom OpenAI Score |  |  |  |  |  |  |
| Mean (± SD) | .927 (± .015) | .943 (± .015) | .945 (± .012) | .950 (± .009) | .953 (± .012) | .952 (± .011) |
| Significance |  | p = 0.001 | p < 0.001 | p < 0.001 | p < 0.001 | p < 0.001 |
| Statistical testing is based on pairwise comparison (Mann-Whitney U Test) between each experimental setting and the baseline. | | | | | | |

Figure 1: Distribution of quantitative similarity metrics (A. BLEU Score; B. ROUGE-L-F1; C. METEOR Score; D. Custom OpenAI Score) across all experiments for all questions, for text-based questions, for table-based questions, and the clinical scenarios.

**Supplementary Note 3: Supplementary Analysis**

We drafted 7 representative questions representing the major section of the guidelines text. All the questions are structured to test reasoning and comprehension from both the main text and non-text sources. We carried out an ablation study in line with what was reported in the main text. Each answer was graded with a score of 1 if the text contained completely accurate information or 0 otherwise. Two expert hepatologists (M.G. and D.L.S.) manually graded each response for accuracy, defined as the presence of totally accurate information based on the guidelines. Disagreements in grading were solved by consensus between the two graders. We repeated the query 5 times each for the 7 questions for each experimental setting and reported the proportion of accurate responses.

| Table 3. We drafted 7 questions that specifically refer to information about management recommendations from text and non-text sources. | |
| --- | --- |
| List of Questions Asked Across Experiments | |
| 1 | What are the recommendations for patients with HCV taking DAAs who have missed DAAs treatment for 3 days? |
| 2 | What are the recommended DAA treatment regimens for adults patients without liver cirrhosis who have previously failed a sofosbuvir based regimen? |
| 3 | What are the recommended DAA treatment regimens for adult patients without liver cirrhosis with recurrent HCV post-kidney transplantation? |
| 4 | What are the recommendations for patients with HCV taking DAAs who have missed DAA treatment for 8 days? |
| 5 | When is a patient with chronic HCV infection eligible for a simplified HCV treatment algorithm? |
| 6 | What are the recommended treatment regimens for pediatric patients (> 3 years of age) with compensated cirrhosis? |
| 7 | What is the recommended screening interval for patients who inject drugs and what are the target counseling strategies to reduce HCV transmission? |

| Table 4. Complete Prompt-Engineering report for all experiments. |
| --- |
| Prompt-Engineering |
| Baseline |
| No prompt was provided, with the exception of the clinical question. |
| Experiment 1 |
| This is a context-based interaction. Based on the following context {Guidelines Text}, answer the following question: {Query}. |
| Experiment 2 |
| This is a context-based interaction. Based on the following context {Guidelines Text} and {Tables .csv Files}, answer the following question: {Query}. |
| Experiment 3 |
| This is a context-based interaction. Based on the following context {Guidelines Text*}, answer the following question: {Query}.  **Tables were converted into lists.* |
| Experiment 4 |
| This is a context-based interaction. Based on the following context {Guidelines Text} that has been formatted according to the following {Instructions**}, answer the following question: {Query}.  ***Instructions:*  These are a series of instructions on how to interpret the text structure:   - Paragraph Title: Recognize this as the main topic or the section header. It gives context to the information that follows. - Paragraph Recommendations: you will find bullet-point directives or guidelines that are meant to be actionable items. Each bullet point is a discrete recommendation with a grading or priority level indicated by the letter and number in parentheses (e.g., A1), which is crucial for understanding the recommendation's weight. - Paragraph Text: This provides the evidence for what is included in the paragraph recommendation.   Besides, you will find in the Text references to Tables that are reported alongside the text and reported as lists. |
| Experiment 5 |
| This is a context-based interaction. Based on the following context {Guidelines Text} that has been formatted according to the following {Instructions*} and a series of {Question-Answer Pairs**} that are examples on how you should provide answers, answer the following question: {Query}.  ****Instructions:*  These are a series of instructions on how to interpret the text structure:   - Paragraph Title: Recognize this as the main topic or the section header. It gives context to the information that follows. - Paragraph Recommendations: you will find bullet-point directives or guidelines that are meant to be actionable items. Each bullet point is a discrete recommendation with a grading or priority level indicated by the letter and number in parentheses (e.g., A1), which is crucial for understanding the recommendation's weight. - Paragraph Text: This provides the evidence for what is included in the paragraph recommendation.   Besides, you will find in the Text references to Tables that are reported alongside the text and reported as lists.  **Question-Answer Pairs:  {"Q":" What is the recommended approach for implementing universal HCV screening?", "A": "Universal HCV screening is recommended for all adults aged ≥18 years. This includes at least once in a lifetime screening, and for pregnant persons during each pregnancy, except in settings where HCV prevalence is <0.1%."},  {"Q": "How has the COVID-19 pandemic affected HCV screening and treatment?" , "A": "The COVID-19 pandemic significantly reduced the level of HCV testing and adversely affected the initiation of HCV treatment. This underscores the importance of maintaining rigorous, universal HCV screening for case identification and linkage to care."},  {"Q": "What is the new algorithm for managing incomplete DAA adherence?", "A": "The new algorithm for managing incomplete DAA adherence involves immediate restart of DAA therapy and extending treatment duration if necessary, depending on the duration of nonadherence and specific patient factors like genotype 3 infection and compensated cirrhosis."},  {"Q": "What are the recommendations for simplified HCV treatment for treatment-naive adults without cirrhosis?", "A": "Treatment-naive adults without cirrhosis can use a simplified treatment algorithm, which includes either 8 weeks of glecaprevir/pibrentasvir or 12 weeks of sofosbuvir/velpatasvir. Minimal monitoring is required, focusing on DAA adherence and SVR assessment."},  {"Q": "What are the key considerations in the simplified HCV treatment for adults with compensated cirrhosis?", "A": "For adults with compensated cirrhosis, the treatment involves either 8 weeks of glecaprevir/pibrentasvir for genotypes 1-6 or 12 weeks of sofosbuvir/velpatasvir for genotypes 1, 2, 4, 5, or 6. Monitoring for liver injury and regular follow-ups for HCC surveillance are important."},  {"Q": "What changes have been made in initial treatment regimens for HCV in the latest guidelines?", "A": "Significant changes in initial treatment regimens include the shortening of glecaprevir/pibrentasvir therapy to 8 weeks for persons with compensated cirrhosis and the use of sofosbuvir/velpatasvir/voxilaprevir as an alternative regimen for genotype 3 infection with compensated cirrhosis."},  {"Q": "What are the recommended initial treatment options for HCV-infected adults without cirrhosis or with compensated cirrhosis?", "A": "Recommended treatments for these adults include glecaprevir/pibrentasvir for 8 weeks and sofosbuvir/velpatasvir for 12 weeks, among others. Specific regimens may vary based on genotype and other patient factors."},  {"Q": "How is retreatment approached for those who fail DAA therapy?", "A": "Retreatment options depend on the initial treatment regimen and patient's cirrhosis status. Options include sofosbuvir/velpatasvir/voxilaprevir for 12 weeks, glecaprevir/pibrentasvir for 16 weeks, among others, with specific considerations for genotype and prior exposure."},  {"Q": "What are the retreatment recommendations for sofosbuvir-based regimen failure?", "A": "For sofosbuvir-based regimen failure, the recommendation is generally 12 weeks of sofosbuvir/velpatasvir/voxilaprevir, with the addition of weight-based ribavirin for genotype 3 infection with compensated cirrhosis."},  {"Q": "What is the recommended approach for managing glecaprevir/pibrentasvir treatment failure?", "A": "In case of glecaprevir/pibrentasvir treatment failure, the recommended option is retreatment with glecaprevir/pibrentasvir plus sofosbuvir and ribavirin for 16 weeks, or sofosbuvir/velpatasvir/voxilaprevir for 12 weeks."},  {"Q": "How should multiple DAA failures, including sofosbuvir/velpatasvir/voxilaprevir or sofosbuvir plus glecaprevir/pibrentasvir, be managed?", "A" : "For multiple DAA failures, a 16-week course of glecaprevir/pibrentasvir plus sofosbuvir and weight-based ribavirin is recommended, with potential extension to 24 weeks in difficult cases."},  {"Q": "What are the guidelines for retreatment in patients with decompensated cirrhosis?", "A": "For decompensated cirrhosis with DAA-based treatment failure, a 24-week course of either sofosbuvir/velpatasvir plus weight-based ribavirin or ledipasvir/sofosbuvir plus weight-based ribavirin is recommended."},  {"Q": "What is the importance of addressing unique and key populations in HCV management?" , "A": "Addressing the needs of unique and key populations, such as HIV/HCV-coinfected persons, PWID, MSM, and incarcerated persons, is vital for significantly reducing the burden of HCV-related disease and achieving HCV elimination targets."},  {"Q": "What are the recommendations for treating HCV in HIV/HCV coinfected persons?", "A": "Treatment-naive persons living with HIV and HCV are eligible for DAA therapy using a simplified treatment algorithm, with a high rate of success demonstrated in clinical trials."},  {"Q": "How should acute HCV infection be managed?", "A": "Persons with confirmed acute HCV infection should be treated the same as those with chronic HCV infection without awaiting possible spontaneous clearance, using the standard DAA regimens."},  {"Q": "What are the guidelines for HCV screening and treatment during pregnancy?", "A": "Universal hepatitis C screening during pregnancy is recommended. DAA treatment may be considered during pregnancy on a case-by-case basis after discussing potential risks and benefits."},  {"Q": "How is HCV infection managed in children?", "A": "All HCV-infected children and adolescents aged ≥3 years should be treated with an approved DAA regimen regardless of disease severity, with efficacy and safety data largely comparable to adults."},  {"Q": "What are the recommendations for managing HCV after solid organ transplantation?", "A": "Post-transplantation, HCV treatment is recommended with various DAA regimens, considering the type of organ transplanted and the patient's cirrhosis status. Drug–drug interactions unique to the posttransplant setting should be addressed."},  {"Q": "How is recurrent HCV infection post liver and kidney transplantation treated?", "A": "Recurrent HCV infection post liver and kidney transplantation is effectively treated with a 12-week course of glecaprevir/pibrentasvir or sofosbuvir/velpatasvir, showing high rates of SVR12."},  {"Q": "What is the approach to treating HCV-uninfected transplant recipients receiving organs from HCV-viremic donors?", "A": "HCV treatment should be initiated as early as possible post-transplantation for HCV-uninfected recipients receiving organs from HCV-viremic donors, with glecaprevir/pibrentasvir or sofosbuvir/velpatasvir being recommended regimens."},  {"Q": "What are the outcomes and processes in transplantation using HCV-viremic donor grafts in HCV-seronegative recipients?", "A": "Transplantation using HCV-viremic donor grafts in HCV-seronegative recipients has shown encouraging outcomes, with comparable survival rates and effective HCV treatment post-transplantation."},  {"Q": "How should HCV infection be managed in people who inject drugs (PWID)?", "A": "Annual HCV testing is recommended for PWID with ongoing IDU. HCV treatment demonstrates high SVR12 rates in this population, and active or recent drug use is not a contraindication to treatment."},  {"Q": "What are the guidelines for managing HCV in men who have sex with men not living with HIV?", "A": "MSM not living with HIV should undergo HCV testing at HIV PrEP initiation and at least annually thereafter. They should be counseled about HCV transmission risks and tested for HCV RNA post-successful treatment or spontaneous clearance."},  {"Q": "What is the recommended approach for managing HCV in correctional settings?", "A": "Opt-out HCV testing consisting of HCV antibody testing followed by confirmatory HCV RNA testing is recommended in correctional settings. DAA treatment should be provided when feasible, with linkage to community healthcare upon release."},  {"Q": "What are the initial treatment recommendations for HCV-infected pediatric patients without cirrhosis or with compensated cirrhosis?", "A": "For pediatric patients, recommended treatments include glecaprevir/pibrentasvir for 8 weeks (genotypes 1-6) and sofosbuvir/velpatasvir for 12 weeks (genotypes 1-6), depending on the genotype and cirrhosis status."},  {"Q": "What are the retreatment options for pediatric patients with prior HCV treatment failure?", "A": "Options include Glecaprevir/Pibrentasvir for 8-16 weeks depending on genotype and cirrhosis status, and Sofosbuvir/Velpatasvir for 12 weeks for genotypes 1–6."},  {"Q": "What is recommended for treating recurrent HCV post liver transplant without cirrhosis?", "A": "Glecaprevir/Pibrentasvir or Sofosbuvir/Velpatasvir for 12 weeks are recommended for genotypes 1–6."},  {"Q": "What is the treatment approach for recurrent HCV post liver transplant with compensated cirrhosis?", "A": "Sofosbuvir/Velpatasvir for 12 weeks for genotypes 1–6 is recommended."},  {"Q": "What are the treatment recommendations for recurrent HCV post kidney transplant?", "A": "For genotypes 1–6, Glecaprevir/Pibrentasvir for 12 weeks is recommended, both for patients without cirrhosis and with compensated cirrhosis."},  {"Q": "How should HCV-uninfected recipients of liver grafts from HCV-viremic donors be treated?", "A": "Treatment should start within the first 2 weeks post-transplant, preferably within the first week, with either Glecaprevir/Pibrentasvir or Sofosbuvir/Velpatasvir for 12 weeks."},  {"Q": "What is the treatment for HCV-uninfected recipients of non-liver solid organs from HCV-viremic donors?", "A": "An 8-week course of Glecaprevir/Pibrentasvir or a 12-week course of Sofosbuvir/Velpatasvir is recommended, initiated immediately pre-transplant or day 0 post-transplant."},  {"Q": "What is the recommendation for annual HCV testing in PWID?", "A": "Annual HCV RNA testing is advised for PWID with ongoing injection drug use, regardless of prior HCV status."},  {"Q": "What is the recommended frequency of HCV testing for MSM not living with HIV on PrEP?", "A": "HCV testing is recommended at HIV PrEP initiation and at least annually thereafter."},  {"Q": "What is the approach to HCV testing and treatment in correctional settings?", "A": "Opt-out HCV testing is recommended in jails and prisons, with treatment provided according to AASLD–IDSA guidance."},  {"Q": "How important is adherence to treatment in achieving successful outcomes in HCV therapy?", "A": "Adherence is critical. Incomplete adherence, especially for extended periods, can lead to reduced efficacy of the treatment and may necessitate extending the treatment duration or considering retreatment strategies."},  {"Q": "What are the key considerations for managing HCV in resource-limited settings?", "A": "In resource-limited settings, emphasis is placed on utilizing simplified treatment algorithms, prioritizing high-risk populations, and implementing cost-effective strategies for screening and treatment."},  {"Q": "When is testing for resistance-associated substitutions (RAS) recommended in HCV treatment?", "A": "RAS testing is particularly important for certain genotypes, like genotype 3, and in cases of treatment failure, to guide the selection of an effective retreatment regimen."},  {"Q": "How is HCV treatment modified in patients with renal impairment?", "A": "Treatment choice and duration may be adjusted in patients with renal impairment, considering the safety and efficacy profiles of DAAs in this population."},  {"Q": "What is the approach to treating HCV patients with co-infections such as HIV or HBV?", "A": "Co-infected patients require careful selection of treatment regimens to manage drug-drug interactions and address the specificities of each infection concurrently."},  {"Q": "Are there special considerations for treating elderly patients with HCV?", "A": "In elderly patients, factors like comorbidities, polypharmacy, and renal function must be considered to ensure safe and effective treatment."},  {"Q": "How does alcohol consumption impact HCV treatment and disease progression?", "A": "Alcohol use can accelerate liver disease progression in HCV-infected patients and may affect treatment adherence. Counseling on reducing or abstaining from alcohol is important."},  {"Q": "What are the key parameters to monitor for assessing the efficacy of HCV treatment?","A": "Monitoring includes assessing quantitative HCV RNA levels at specified intervals during and after treatment to confirm virologic response and cure."},  {"Q": "What does post-treatment follow-up involve for patients cured of HCV?", "A": "It includes monitoring liver function, assessing for liver disease complications in cirrhotic patients, and counseling on lifestyle changes to prevent reinfection."},  {"Q": "How is the risk of HCV reinfection addressed post-cure?", "A": "Patients are counseled on risk reduction, particularly those with ongoing risk factors like injection drug use, and might be monitored with periodic HCV RNA testing."},  {"Q": "What are the considerations for managing HCV in pregnant women?", "A": "While routine HCV screening is recommended in pregnancy, treatment decisions are made on a case-by-case basis, considering the potential risks and benefits of DAAs during pregnancy."},  {"Q": "Are there specific considerations for managing HCV in children?", "A": "Yes, treatment options for children are based on age, weight, HCV genotype, and liver disease status, with DAAs like glecaprevir/pibrentasvir and sofosbuvir/velpatasvir being commonly used."},  {"Q": "How is HCV managed in patients who have undergone liver transplantation?", "A": "Post-transplant HCV management involves careful selection of DAAs to avoid drug interactions with immunosuppressants and to address the specific needs of the transplanted liver."},  {"Q": "What are the major drug interaction concerns in HCV treatment?", "A": "Significant interactions can occur between DAAs and other medications, including HIV antiretrovirals, immunosuppressants, and certain anticoagulants, requiring careful medication reconciliation and adjustment."},  {"Q": "What strategies can improve access to HCV care and treatment?", "A": "Strategies include increasing awareness, simplifying treatment protocols, integrating HCV services into primary care and substance use treatment programs, and leveraging telemedicine."},  {"Q": "Is surveillance for hepatocellular carcinoma recommended in HCV patients post-treatment?", "A": "Yes, particularly in patients with cirrhosis, regular surveillance for HCC is recommended even after achieving SVR."},  {"Q": "How are HCV-infected patients with psychiatric disorders managed?", "A": "These patients require a multidisciplinary approach with close monitoring, potential psychiatric medication adjustments, and support to ensure adherence to HCV treatment."},  {"Q": "What are the considerations for treating HCV in patients with hemoglobinopathies?", "A": "In patients with conditions like thalassemia or sickle cell disease, treatment regimens and durations may need adjustment, and careful monitoring for potential drug interactions or adverse effects is necessary."},  {"Q": "How is HCV treatment approached in patients with renal disease?", "A": "Treatment choice should consider the patient's renal function, with some DAAs being more suitable for patients with reduced renal function or on dialysis."},  {"Q": "What strategies are effective in managing HCV in homeless populations?", "A": "Outreach programs, integration of HCV services into existing support services for the homeless, and simplified treatment protocols can enhance engagement and adherence in this population."} |

Figure 3: Qualitative evaluation of accuracy among all experiments from baseline for all questions. The reported statistical difference is obtained by comparing each experiment to the baseline.

Figure 4: Distribution of text-similarity metrics across all experiments for all questions for Northern America Guidelines). Abbreviations: Exp: experiment.

| Table 5.: In evaluating text generation similarity, we employed automatic metrics that scrutinized critical facets like word overlap and semantic coherence by comparing LLM-generated answers to our human-expert provided gold-standard. | | | | | | |
| --- | --- | --- | --- | --- | --- | --- |
| Metrics | **Baseline** | **Experiment 1** | **Experiment 2** | **Experiment 3** | **Experiment 4** | **Experiment 5** |
| BLEU score |  |  |  |  |  |  |
| Mean (± SD) | .019 (± .014) | .069 (± .080) | .058 (± .074) | .156 (± .162) | .138 (± .099) | .127 (± .070) |
| Significance |  | p < 0.001 | p = 0.008 | p < 0.001 | p < 0.001 | p < 0.0001 |
| ROUGE-LCS F1 |  |  |  |  |  |  |
| Mean (± SD) | .185 (± .042) | .315 (± .126) | .296 (± .102) | .434 (± .153) | .424 (± .126) | .388 ± .097) |
| Significance |  | p < 0.0001 | p < 0.001 | p < 0.001 | p < 0.001 | p < 0.001 |
| METEOR score F1 |  |  |  |  |  |  |
| Mean (± SD) | .280 (± .055) | .397 (± .117) | .366 (± .117) | .520 (± .126) | .511 (± .097) | .417 ± .117) |
| Significance |  | p < 0.001 | p = 0.002 | p < 0.001 | p < 0.001 | p < 0. 001 |
| OpenAI Score |  |  |  |  |  |  |
| Mean (± SD) | .928 (± .017) | .953 (± .016) | .951 (± .018) | .965 (± .012) | .964 (± .015) | .961 ± .017) |
| Significance |  | p < 0.001 | p < 0.01 | p < 0.001 | p < 0.001 | p < 0.001 |
| Statistical testing is based on pairwise comparison (Mann-Whitney U Test) between each experimental setting and the baseline. | | | | | | |
